# Augmented State-Space Modeling and Control of Latent Arousal States Under Inhibitory and Excitatory Conditions

**DOI:** 10.1109/OJEMB.2026.3672470

**Published:** 2026-03-10

**Authors:** Hamid Fekri Azgomi, Anan Yaghmour, Rose T. Faghih

**Affiliations:** Electrical and Computer Engineering DepartmentUniversity of Houston 14743 Houston TX 77004 USA; Department of Neurological SurgeryUniversity of California San Francisco 8785 San Francisco CA 94143 USA; Electrical and Computer Engineering DepartmentUniversity of Houston 14743 Houston TX 77004 USA; X10e Technology Company Mountain View CA 94049 USA; Department of Biomedical Engineering, Tandon School of EngineeringNew York University 5894 New York NY 11201 USA

**Keywords:** Adaptive control, Cognitive arousal regulation, Robust control, Skin conductance, State-space

## Abstract

*Goal:* In modern high-stress environments, effectively regulating cognitive arousal, through enhancement to boost engagement or inhibition to manage excessive stress, is essential for maintaining mental well-being and optimizing human performance. Hence, this study extends existing state-space models by integrating time-varying parameters and disturbance inputs for enhanced representation of arousal dynamics inferred from skin conductance. *Methods:* We augmented nominal models with time-varying parameters, then developed a recursive Bayesian estimator for state tracking. Simulation-based validation was performed using skin conductance data from six participants, drawn from an experimental dataset of noninvasive wrist-worn physiological recordings acquired during cognitive stress and relaxation tasks. Adaptive and robust control architectures were designed for closed-loop regulation of latent arousal states. *Results:* Simulations based on experimental data showed that both controllers outperformed static methods. On average, under inhibitory and excitatory conditions, the adaptive controller achieved average RMSE reductions of 26.9 and 51.6, respectively, while the robust controller achieved reductions of 16.0 and 23.4. In complex multi-step tracking, the adaptive controller reduced average RMSE by 33.7 and control effort by 18.5; similarly, the robust controller reduced RMSE by 32.6 and control effort by 15.1. *Conclusion:* These findings demonstrate that adaptive and robust control strategies can reliably manage dynamic arousal regulation, offering potential for real-world neuroadaptive systems supporting human performance and well-being.

## I. Introduction

In today's demanding environments, the effective management of cognitive arousal is essential for preserving mental health and enhancing human performance. Although cognitive arousal cannot be directly measured, it can be inferred from autonomic nervous system-mediated physiological indicators, such as electrodermal activity, heart rate variability, respiration, and pupil dynamics, which reflect changes in arousal states [1]. The rapid advancement and widespread adoption of wearable technologies have revolutionized health monitoring and management by providing continuous streams of physiological data, offering exceptional insights into the body's internal states. Mathematical models play essential roles in translating these measurements into meaningful information about latent internal states [2]. By linking observable physiological signals to hidden internal states, these models enable not only the tracking but also the real-time regulation of such states [3], [4], [5], [6]. Despite recent advancements, the practical implementation of systems to track and regulate latent internal states faces significant challenges, including sensor noise, external disturbances, and inherent variability in physiological systems both between and within individuals [7]. These factors complicate the scalability and effectiveness of control methodologies in real-world applications, underscoring the need for more robust, adaptable modeling and control strategies.

Electrodermal activity (EDA) is a noninvasive physiological measure of autonomic nervous system activity, quantified via changes in skin conductance and measurable through wearable devices, making it a reliable indicator of mental arousal and cognitive stress [8], [9], [10], [11]. The autonomic nervous system (ANS) controls the body's response to stress, with changes in ANS activity reflected in physiological signals like heart rate, breathing, and skin conductivity [12], [13], [14]. When the brain reacts to stress, sweat gland activity is modulated via the ANS, leading to detectable changes in EDA via wrist-worn sensors. Mathematical models, particularly state-space representations, are effectively used to translate the fluctuation in EDA signals into internal cognitive arousal state [6], [15], [16], [17]. These models link measured EDA signals to hidden arousal states, enabling their estimation and real-time regulation. Utilizing control system algorithms, it is possible to close the loop between monitoring and action, employing feedback control to maintain arousal states within target ranges [3], [4]. However, current models often overlook critical sources of variability, including inter- and intra-subject physiological differences, time-varying autonomic dynamics, and unmodeled external disturbances, thereby limiting the real-world applicability and reliability of existing control systems.

Several feedback control strategies have been proposed for regulating uncertain and disturbed dynamical systems, including PID-based, fractional-order, and disturbance-rejection controllers, many of which have demonstrated effectiveness in industrial and process-control applications [18], [19], [20], [21]. More recent efforts have extended these approaches to fractional-order and nonlinear formulations, with applications ranging from biochemical reactor control to biomedical-inspired problems such as chemotherapy dose scheduling [22], [23]. Despite their practical appeal, these methods are typically developed under fixed or limited-adaptation model assumptions and do not explicitly account for time-varying physiological parameters or principled uncertainty quantification. Such limitations become particularly consequential in neurophysiological arousal regulation, where pronounced inter- and intra-subject variability, nonstationary dynamics, and stringent safety requirements limit the applicability of static or heuristically tuned control architectures.

To address these challenges, we extend nominal state-space models [3], [4] to incorporate time-varying parameters and structured disturbance inputs, enabling a more faithful representation of physiological variability. Building on this augmented modeling framework, we develop recursive Bayesian estimators to jointly infer latent arousal states and evolving system parameters in real time. Leveraging these probabilistically informed estimates, we design adaptive and robust control architectures within a linear-quadratic regulator (LQR) framework, yielding state-feedback policies that are optimal with respect to a quadratic performance criterion conditioned on the inferred system dynamics [24]. Through continuous monitoring and real-time feedback, the resulting closed-loop system regulates estimated arousal states under both inhibitory and excitatory conditions while maintaining stability and performance in the presence of uncertainty. Simulation studies based on experimental data further validate the proposed framework, highlighting its potential for safe, scalable, and principled neurobehavioral regulation in therapeutic and performance-oriented applications.

## II. Materials and Methods

**Fig. 1.**
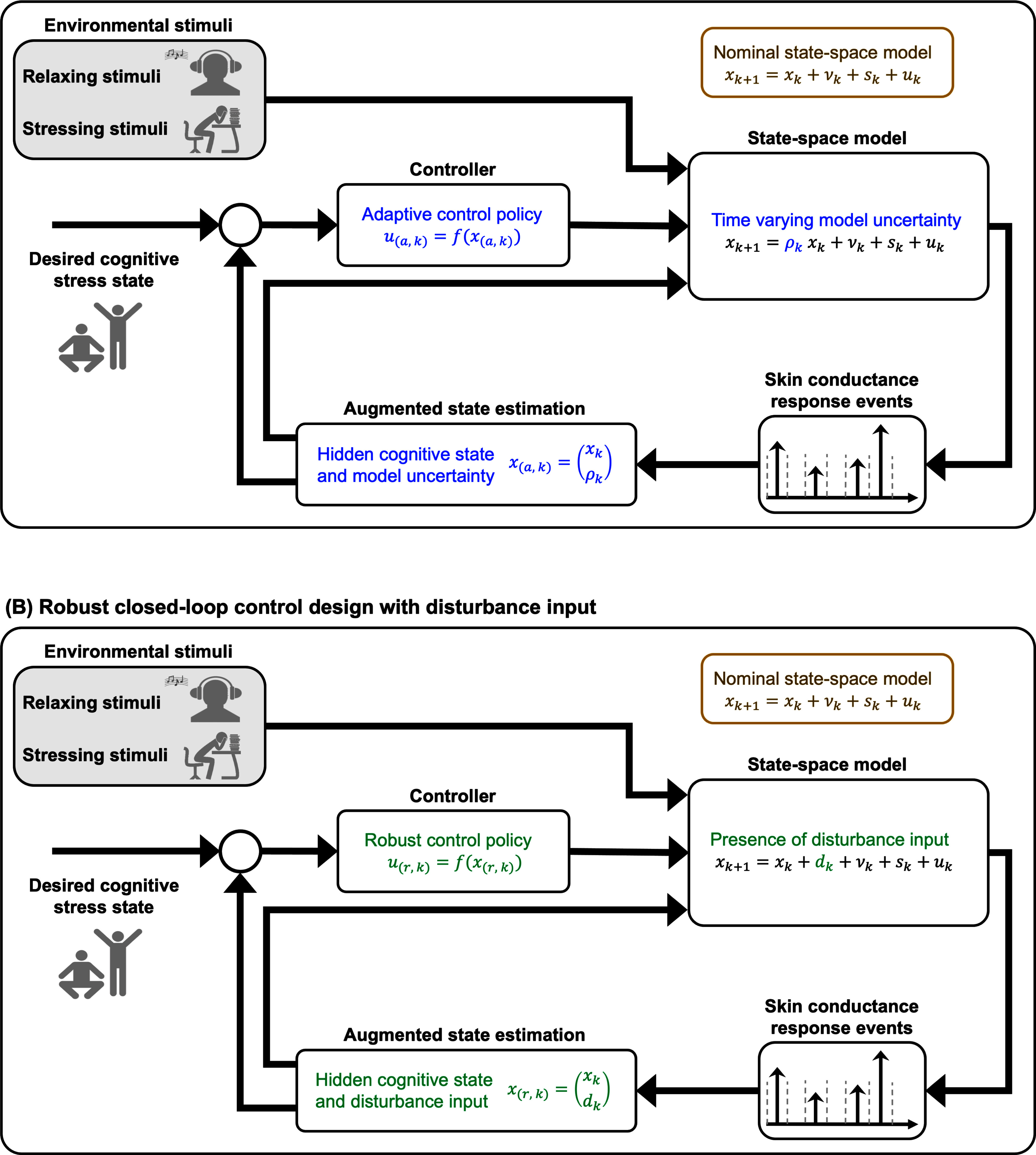
Closed-Loop Control Design for Cognitive Stress State Regulation. Panels (a) and (b) illustrate the schematics of the adaptive and robust closed-loop control systems, respectively, designed to regulate the cognitive stress state within a simulation environment based on experimental data. Relaxing and stress-inducing stimuli are simulated to produce hidden cognitive stress state fluctuations. In panel (a), the nominal state-space model is augmented by incorporating time-varying model parameters to capture physiological variability. In panel (b), a disturbance input is included to account for additional external variability. Within each system, simulated skin conductance response (SCR) events are used as observations to track the augmented model dynamics using a recursive Bayesian state estimator. Optimal control strategies are then designed to maintain the estimated cognitive stress state within desired ranges through closed-loop feedback mechanisms.

Fig. 1 presents an overview of our proposed framework.

### A. Human Brain Stimulus-Response Model

The simulation model used in this study was derived from experimental human-subject data collected under Institutional Review Board approval (UTD IRB #12-29) at the University of Texas at Dallas and is publicly available as the Non-EEG Dataset for Assessment of Neurological Status through the PhysioNet database [25], [26]. The dataset comprises recordings from healthy adult participants who were exposed to a structured sequence of relaxation, physical stress, cognitive stress, and emotional stress tasks, with multiple relaxation periods interleaved throughout the protocol.

In the present work, we focus specifically on the cognitive stress and relaxation sessions, which represent high- and low-arousal extremes and are most informative for modeling and regulating latent cognitive arousal states. Cognitive stress consisted of a combined arithmetic task (counting backward by sevens for three minutes) and a Stroop task (two minutes), while relaxation periods involved seated rest with calming auditory stimuli. Among the multimodal physiological signals available in the dataset, we exclusively used electrodermal activity (EDA) measurements from the Affectiva Q Curve wearable device, as EDA provides a direct and reliable proxy for sympathetic nervous system arousal. Consistent with prior studies using this dataset [3], [4], [27], analysis was restricted to six participants whose EDA recordings exhibited minimal motion artifacts, no signal saturation, and stable sensor amplification characteristics. Participants with excessive noise, missing segments, or pronounced motion-induced contamination were excluded to ensure reliable system identification and control validation. All signals were assumed to be pre-segmented according to task labels provided in the dataset, and no additional preprocessing beyond artifact-based participant exclusion was performed. The resulting EDA time series were used to construct a simulation environment for estimating and regulating latent cognitive arousal states via augmented state-space modeling and closed-loop control. Additional details on the experimental protocol and baseline modeling assumptions can be found in [3], [4].

Using the simulation framework developed in [3], [4], we begin with a nominal state-space model that relates a latent cognitive stress state to observed skin conductance measurements. This nominal formulation serves as the baseline for subsequent model extensions that incorporate parameter uncertainty and disturbance inputs. Specifically, in the absence of model uncertainty and external disturbances, the latent stress-related state dynamics are represented using a first-order state-space model that captures changes in skin conductance signals, as described in [3], [4]

\begin{align*}
x_{k+1} = x_{k} + s_{k} + \nu _{k} + u_{k}, \tag{1}
\end{align*}

where $x_{k}$ corresponds to the hidden cognitive stress-related state, $s_{k}$ stands for the environmental stimuli, and $\nu _{k} \sim \mathcal {N}(0,\,\sigma ^{2}_\nu)$ represents the process noise. $u_{k}$ is the control signal to regulate the simulated stress-related state. It should be noted that $s_{k}$ in (1) is only for simulation purposes. In the experimental implementation, the internal cognitive stress state is influenced by the actual environment. Additional details of modeling the environmental stimuli are presented in [4]. In the literature, the dynamics of hidden neural states are frequently modeled as random walks and first-order autoregressive with extra input (ARX) models [17], [28]. We utilize the same family of models to capture the evolution of the cognitive stress-related state through time. While (1) captures the evolution of the hidden state, we also require an observation model that links this latent variable to measurable skin conductance response (SCR) events and continuous skin conductance features. To model the observation signal, we assume the occurrence of SCR events, $n_{k}$, follows a Bernoulli distribution with the probability function

\begin{align*}
P(n_{k} | x_{k})= q_{k} ^{n_{k}} (1- q_{k})^{1-n_{k}}, \tag{2}
\end{align*}

where the probability $q_{k}$ is linked to the stress state $x_{k}$, via the Sigmoid function [29]

\begin{align*}
q_{k} = \frac{1}{1+ e^{-(\gamma + x_{k})}}, \tag{3}
\end{align*}

where $\gamma$ is the person-specific baseline parameter that should be determined. Similar to [3], [4], we first assume $x_{0}$ approximately equals zero. We then calculate $\gamma$ based on the average probability of receiving SCR in the whole data. According to (3), with an increase in the levels of the cognitive stress state, the probability of receiving the SCR events is also increased. To enhance the model, we incorporate additional information presented in the skin conductance signal and employ continuous features as well [3], [16]. Thus, we assume there is a linear relationship between the internal cognitive stress state, $x_{k}$, and the tonic component of the skin conductance signal

\begin{align*}
r_{k} = \rho _{0} + \rho _{1} x_{k} + \zeta _{k}, \tag{4}
\end{align*}

where $r_{k}$ is assumed to be the *log* transformation of the continuous-valued observation associated with the tonic component of the skin conductance signal. $\rho _{0}$ and $\rho _{1}$ are constant values derived by the offline expectation maximization algorithm [16]. $\zeta _{k} \sim \mathcal {N} (0,\sigma _\zeta ^{2})$ is measurement noise with variance $\sigma _\zeta ^{2}$.

Building on this nominal model, we next consider two sources of variability that are critical for realistic arousal regulation: (i) uncertainty in model parameters due to physiological variability, and (ii) disturbance inputs representing unmodeled external influences. In the first scenario, we focus on designing an adaptive control system that manages model uncertainties. In the second scenario, we develop a robust control scheme to mitigate the effects of undesired disturbance inputs within the modeled dynamics.

### B. State-Space Modeling in the Presence of Time-Varying Model Uncertainty

To incorporate model uncertainty in state-space formulation (1), we relax the imposed time-invariant condition [24]

\begin{align*}
x_{k+1} &= \rho _{k} x_{k} +u_{k}+s_{k}+ \nu _{k}, \tag{5}
\end{align*}

where uncertainty in model parameter $\rho _{k}$ is modeled as a random walk process

\begin{align*}
\rho _{k} &= \rho _{k-1}+\epsilon _{k} \ \text{ and} \quad \epsilon _{k}\sim \mathcal {N}(0,\sigma ^{2}_\epsilon). \tag{6}
\end{align*}

To jointly estimate the latent state and the uncertain parameter (i.e., $\rho _{k}$ and $x_{k}$), we define an augmented state vector that captures both quantities, enabling recursive Bayesian estimation of the expanded system based on the observation $\mathbf{y}_{k}= \begin{pmatrix}n_{k} \\
 r_{k} \end{pmatrix}$. We derive the two-dimensional augmented state vector

\begin{align*}
\mathbf{x}_{a,k} = \begin{pmatrix}x_{k} \\
\rho _{k}\end{pmatrix} = \begin{pmatrix}\bf x_{a,k}^{(1)} \\
\mathbf{x}_{a,k}^{(2)} \end{pmatrix}. \tag{7}
\end{align*}

Therefore, the augmented system's dynamics is

\begin{align*}
\mathbf{x}_{a,k+1} &= \begin{pmatrix}\bf x_{a,k}^{(2)} & 0\\
0&1\end{pmatrix} \mathbf{x}_{a,k}+ \begin{pmatrix}1\\
0\end{pmatrix}u_{k}+{\mathbf{w}}_{a,k} \\
&\triangleq f_{a} (\mathbf{x}_{a,k},u_{k})+\mathbf{w}_{a,k} \tag{8}
\end{align*}

where $\mathbf{w}_{a,k}=\begin{pmatrix}\nu _{k}\\
 \epsilon _{k}\end{pmatrix}$, is a Gaussian random vector with zero mean vector, and covariance matrix $K_{a}=\begin{pmatrix}\sigma ^{2}_{\nu }&0\\
0&\sigma ^{2}_{\epsilon } \end{pmatrix}$. The proposed recursive Bayesian estimator consists of the prediction and update steps. The prediction step relies on a recursive probabilistic model for the time evolution of the augmented states. The update step utilizes a probabilistic observation model relating the hidden cognitive stress-related arousal state to the SCR events time and continuous feature.

*Prediction step:*

\begin{align*}
\mathbf{x}_{a,k|k-1} & = f_{a}(\mathbf{x}_{a,k-1|k-1},u_{k}) \tag{9}\\
\text{and } {\Sigma }_{a,k|k-1} & = \mathbf{F}_{a,k-1} {\Sigma }_{a,k-1|k-1} \mathbf{F}_{a,k-1}^{\prime }+\mathbf{K}_{a}, \tag{10}
\end{align*}

where $(\cdot)^{\prime }$ denotes the transpose operation and $\mathbf{F}_{a,k-1}=[\frac{\partial f_{a}}{\partial \mathbf{x}_{a}}]_{ \mathbf{x}_{a,k-1|k-1}}$. $ \mathbf{x}_{a,k|k-1}$ and ${\Sigma }_{a,k|k-1}$ stand for the mean and the covariance of the prediction of $\mathbf{x}_{a,k}$ given all the previous $(k-1)$ observations (i.e., $\mathbf{y}_{1:k-1}$ at time step $k$), respectively.

*Update step:*

\begin{align*}
\mathbf{x}_{a,k|k} & = \mathbf{x}_{a,k|k-1} + { {\Sigma }}_{a,k|k}\begin{pmatrix}g_{a,k}\\
0\end{pmatrix} \tag{11}\\
\text{and } {\Sigma }_{a,k|k} &= {\Sigma }_{a,k|k-1} +\begin{pmatrix}\left[\frac{\partial {g}_{a,k}}{\partial \mathbf{x}_{a,k}^{(1)}}\right]_{\mathbf{x}_{a,k}^{(1)}=\mathbf{x}_{a,k|k-1}^{(1)}}&0\\
0&0\end{pmatrix}, \tag{12}
\end{align*}

where

\begin{align*}
{g}_{a,k} & = \left[\frac{\partial \log \left(p(\mathbf{y}_{k}|\mathbf{x}_{a,k}^{(1)}) \right)}{\partial \mathbf{x}_{a,k}^{(1)}}\right]_{\mathbf{x}_{a,k}^{(1)} = \mathbf{x}_{a,k|k-1}^{(1)} }. \tag{13}
\end{align*}

Thus, we estimate the augmented dynamic $\mathbf{x}_{a,k}$ in real time.

### C. Adaptive Feedback Controller Design

With the augmented states estimated in real time, we next turn to the design of an adaptive feedback controller that can regulate the latent arousal state despite model uncertainty. We pursue the goal of designing an adaptive feedback controller to derive the control input $u_{k}$ based on the real-time estimate of the latent state $x_{k}$ in augmented state $\mathbf{x}_{a,k}$ above (7) [24]. This includes finding the $u_{k}$ to optimize a quadratic cost function defined as

\begin{align*}
J_{a}&= \sum _{k=0}^{\infty} Q_{a}(x_{k}-x^{*}) ^{2}+R_{a}(u_{k}-{{u}}^{*})^{2}, \tag{14}
\end{align*}

where $Q_{a}$ and $R_{a}$ are positive weight matrices. $x^{*}$ and $u^{*}$ are the target state and the corresponding control value. By fixing the time-varying parameter at its current estimate (i.e., $ \rho _{k} = \mathbf{x}_{a,k|k}^{(2)}$), ignoring environmental stimuli, and given the system reaches the steady state, we derive $u^{*} = x^{*}(1-\mathbf{x}_{a,k|k}^{(2)})$ (5).

Fixing the cognitive stress-related arousal state dynamics parameters at their current estimate changes (14) to a non-zero set-point LQR problem [30], [31]. To convert it to a traditional LQR formulation, where the control goal is to derive the state close to the origin, we let $\tilde{x}_{k}=x_{k}-x^{*},\, \tilde{u}_{k}=u_{k}-u^{*}_{k}$. Now, the optimal $\tilde{u}_{k}$ for the classical LQR problem is simply a linear feedback control as

\begin{align*}
\tilde{u}_{k}= -l_{a,k} \tilde{x}_{k}, \tag{15}
\end{align*}

where $l_{a,k}$ is a scalar feedback gain derived as

\begin{align*}
l_{a,k} = \frac{\rho _{k} P_{a,k}}{P_{a,k}+R_{a}}, \tag{16}
\end{align*}

where $P_{a,k}$ is the solution of the algebraic Riccati equation in the discrete form [30]

\begin{align*}
\rho ^{2}_{k} P_{a,k}+\frac{\rho ^{2}_{k} P_{r,k}^{2}}{P_{a,k}+R_{a}}+Q_{a}=P_{a,k}. \tag{17}
\end{align*}

Thus, the optimal control signal $u_{k}$ will be drived as $u_{k}={u}^{*} - l_{a,k}(x_{k}-x^{*})$.

In parallel, we also consider the case in which exogenous disturbances affect the system dynamics. To capture this scenario, the nominal model is further extended with an additional disturbance term.

### D. State-Space Modeling in the Presence of Disturbance Input

In this part, we consider an additional term to the nominal dynamical system as a disturbance input. The nominal system (1) expands as

\begin{align*}
x_{k+1} &= x_{k} +u_{k}+s_{k}+ \nu _{k}+ d_{k}, \tag{18}
\end{align*}

where the disturbance input $d_{k}$ is modeled as a random walk process

\begin{align*}
d_{k}&= d_{k-1}+\omega _{k}\quad \text{ and } \omega _{k}\sim \mathcal {N}(0,\sigma ^{2}_{\omega _{k}}). \tag{19}
\end{align*}

As in the adaptive framework, we jointly represent the latent state and disturbance in an augmented state vector, enabling simultaneous estimation of both. Concatenating $x_{k}$ and $d_{k}$ into an augmented two-dimensional state vector

\begin{align*}
\mathbf{x}_{r,k} = \begin{pmatrix}x_{k} \\
d_{k}\end{pmatrix} = \begin{pmatrix}\bf x_{r,k}^{(1)} \\
\mathbf{x}_{r,k}^{(2)} \end{pmatrix}. \tag{20}
\end{align*}

we derive the augmented system's dynamics as

\begin{align*}
\mathbf{x}_{r,k+1} &= \begin{pmatrix}1 & 1\\
0&1\end{pmatrix} \mathbf{x}_{r,k}+ \begin{pmatrix}1\\
0\end{pmatrix}u_{k}+{\mathbf{w}}_{r,k} \\
&\triangleq f_{r} (\mathbf{x}_{r,k},u_{k})+\mathbf{w}_{r,k} \tag{21}
\end{align*}

where $\mathbf{w}_{r,k}=\begin{pmatrix}\nu _{k}\\
 \omega _{k}\end{pmatrix}$, is a Gaussian random vector with zero mean vector, and covariance matrix $\mathbf{{K}}_{r}=\begin{pmatrix}\sigma ^{2}_{\nu }&0\\
0&\sigma ^{2}_{\omega } \end{pmatrix}$. In what follows, we derive the prediction and update steps for estimating variables in an augmented system.

*Prediction step:*

\begin{align*}
\mathbf{x}_{r,k|k-1} & = f_{r}(\mathbf{x}_{r,k-1|k-1},u_{k}) \tag{22}\\
\text{and } {\Sigma }_{r,k|k-1} & = \mathbf{F}_{r,k-1} {\Sigma }_{r,k-1|k-1} \mathbf{F}_{r,k-1}^{\prime }+\mathbf{K}_{r}, \tag{23}
\end{align*}

where $(\cdot)^{\prime }$ denotes the transpose operation and $\mathbf{F}_{r,k-1}=[\frac{\partial f_{r}}{\partial \mathbf{x}_{r}}]_{ \mathbf{x}_{r,k-1|k-1}}$. $\mathbf{x}_{r,k|k-1}$ and $ {\Sigma }_{r,k|k-1}$ stand for the mean and the covariance of the prediction of $\mathbf{x}_{r,k}$ given all the previous $(k-1)$ observations (i.e., $\mathbf{y}_{1:k-1}$ at time step $k$), respectively.

*Update step:*

\begin{align*}
\mathbf{x}_{r,k|k} & = \mathbf{x}_{r,k|k-1} + { {\Sigma }}_{r,k|k}\begin{pmatrix}g_{r,k}\\
0\end{pmatrix} \tag{24}\\
\text{and } {\Sigma }_{r,k|k} &= {\Sigma }_{r,k|k-1} +\begin{pmatrix}\left[\frac{\partial {g}_{r,k}}{\partial \mathbf{x}_{r,k}^{(1)}}\right]_{\mathbf{x}_{r,k}^{(1)}=\mathbf{x}_{r,k|k-1}^{(1)}}&0\\
0&0\end{pmatrix}, \tag{25}
\end{align*}

where

\begin{align*}
{g}_{r,k} & = \left[\frac{\partial \log (p(\mathbf{y}_{k}|\mathbf{x}_{r,k}^{(1)})) }{\partial \mathbf{x}_{r,k}^{(1)}}\right]_{\mathbf{x}_{r,k}^{(1)} = \mathbf{x}_{r,k|k-1}^{(1)} }. \tag{26}
\end{align*}

### E. Robust Feedback Controller Design

Once the augmented states are estimated, we design a robust feedback controller to maintain the arousal state within desired bounds while compensating for disturbance inputs. Here, we design a robust feedback controller to derive the control action $u_{k}$ [32]. Similar to adaptive control design, we formulate a quadratic cost function as

\begin{align*}
{ {J}_{r}}&= \sum _{k=0}^{\infty} Q_{r}(x_{k}-x^{*}) ^{2}+R_{r}(u_{k}-{u}^{*})^{2}, \tag{27}
\end{align*}

where $Q_{r}$ and $R_{r}$ are positive weight matrices. $x^{*}$ and $u^{*}$ are the target state and the corresponding control value. By fixing the time-varying parameter at its current estimate (i.e., $ d_{k} = \mathbf{x}_{r,k|k}^{(2)}$), ignoring environmental stimuli, and given the system reaches the steady state, we derive a single linear equation described in (18). Solving it for $u^{*}_{k}$, we derive $u^{*}_{k} = -\mathbf{x}_{r,k|k}^{(2)}$.

To convert it to a traditional LQR formulation, where the control goal is to derive the state close to the origin, we let $\tilde{x}_{k}=x_{k}-x^{*},\, \tilde{u}_{k}=u_{k}-u^{*}$. The optimal $\tilde{u}_{k}$ for the classical LQR problem is simply a linear feedback of the form given

\begin{align*}
\tilde{u}_{k}= -l_{r,k} \tilde{x}_{k}, \tag{28}
\end{align*}

where $l_{r,k}$ is a scalar feedback gain derived as

\begin{align*}
l_{k} = \frac{P_{r,k}}{P_{r,k}+R_{a}}, \tag{29}
\end{align*}

where $P_{r,k}$ is the solution of the algebraic Riccati equation in the discrete form [30]

\begin{align*}
P_{r,k}+\frac{P^{2}_{r,k}}{P_{r,k}+R_{a}}+Q_{a}=P_{r,k}. \tag{30}
\end{align*}

Hence, the optimal control signal $u_{k}$ is derived as $u_{k}={u}^{*} - l_{r,k}(x_{k}-x^{*})$.

## III. Results

**Fig. 2.**
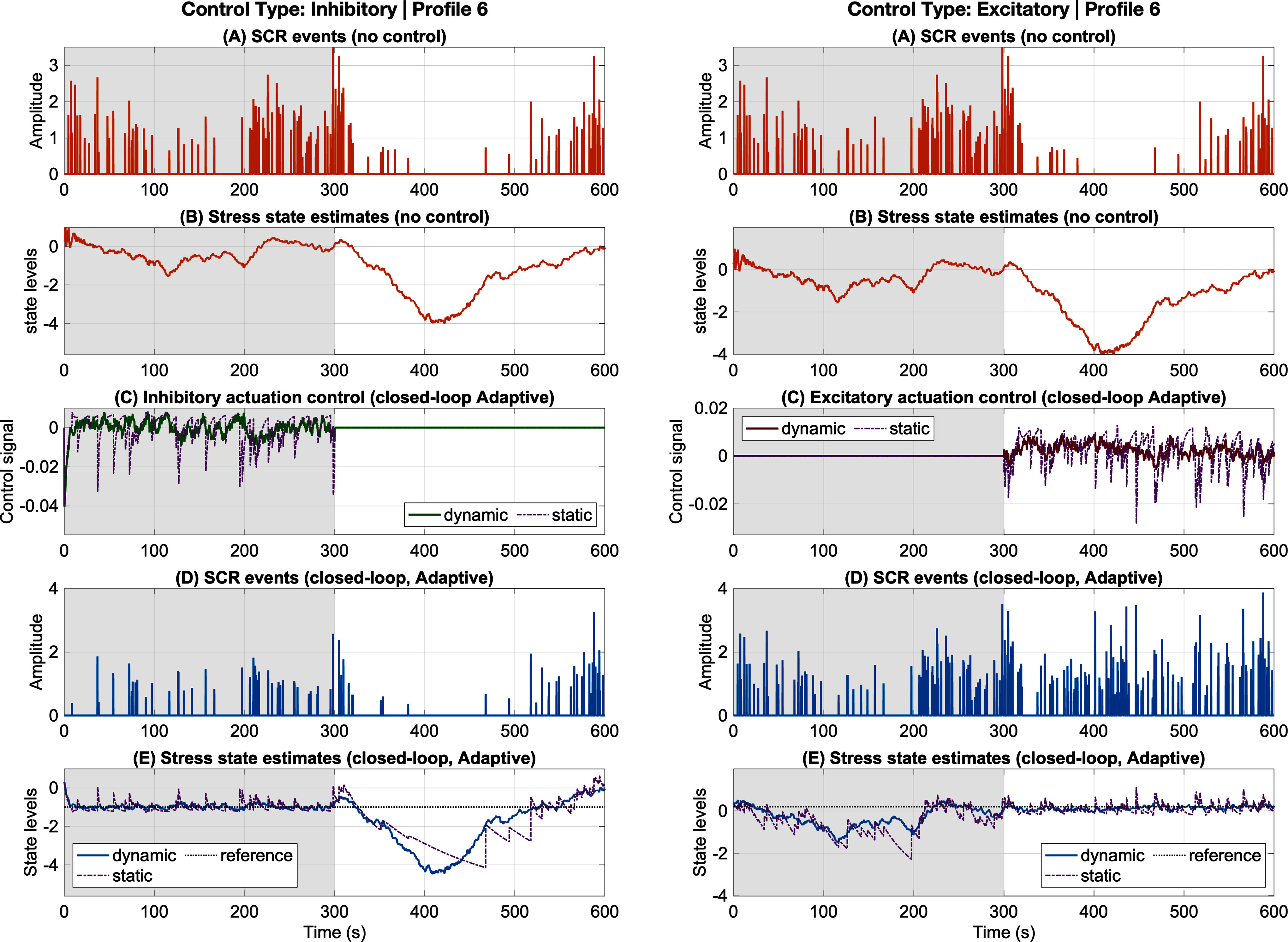
Adaptive inhibition and excitation results (Profile 6). The left and right panels correspond to inhibition and excitation, respectively. In each panel: (a) and (b) show SCR events and estimated stress state under no control; (c) shows the closed-loop dynamic adaptive control input (green for inhibition, red for excitation) together with the static control (purple); (d) shows SCR events under closed-loop adaptive control; and (e) shows the estimated stress state under closed-loop adaptive control (blue) and static control (purple). Grey and white backgrounds denote high- and low-arousal environmental stimuli, respectively.

**Fig. 3.**
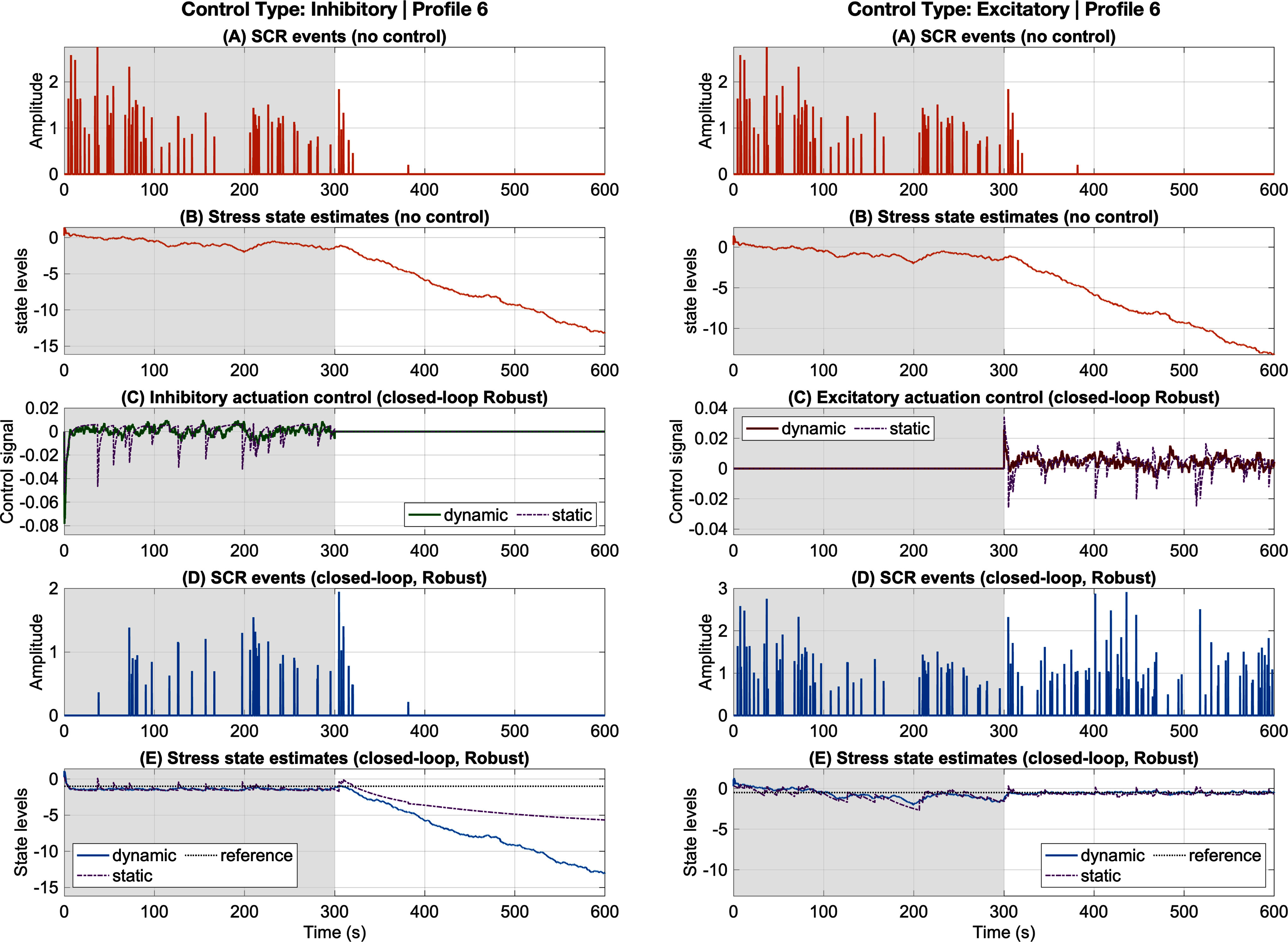
Robust inhibition and excitation results (Profile 6). The left and right panels correspond to inhibition and excitation, respectively. In each panel: (a) and (b) show SCR events and estimated stress state under no control; (c) shows the closed-loop dynamic robust control input (green for inhibition, red for excitation) together with the static control (purple); (d) shows SCR events under closed-loop robust control; and (e) shows the estimated stress state under closed-loop robust control (blue) and static control (purple). Grey and white backgrounds denote high- and low-arousal environmental stimuli, respectively.

We evaluated the performance of the adaptive and robust closed-loop controllers in the simulation environment introduced in [4], in which cognitive arousal states were alternately driven by stress-inducing and relaxing stimuli. Results are presented for both inhibitory and excitatory control classes, with representative outcomes shown for one participant (Figs. 2, 3). Full simulation results for all participants are included in the supplementary material (Figures S1 S10). To ensure quantitative assessment beyond individual examples, we defined objective performance metrics and compared results across multiple conditions.

Two complementary metrics were used to evaluate system performance: (1) the root-mean-square error (RMSE) between the reference signal and the estimated arousal state, which quantifies the accuracy of state regulation, and (2) the total control input, computed as $\sum _{k} |u_{k}|$, which captures the energetic cost of regulation. These metrics jointly capture the fundamental trade-off between regulation accuracy and actuation demand, enabling interpretation of controller behavior not only in terms of tracking performance but also efficiency and robustness across operating conditions. These metrics provide a consistent basis for comparing open-loop versus closed-loop responses, adaptive versus robust models, and inhibitory versus excitatory control. Throughout this work, *open-loop* refers to conditions in which a controller is present but model parameters and disturbance inputs are not estimated as part of the augmented state, whereas *closed-loop* refers to the adaptive and robust implementations that explicitly estimate these quantities to improve tracking of the desired arousal trajectory. Representative trajectories are shown in Figs. 2 and 3, and aggregate performance metrics across all participants are summarized in Table 1.

**TABLE I:** Percentage Changes in Tracking Error (RMSE) and Control Effort Under Dynamic Control Relative to the Static Case

	Adaptive ()						Robust ()					
	Multi-step		Inhibition		Excitation		Multi-step		Inhibition		Excitation	
Profile	RMSE	control	RMSE	control	RMSE	control	RMSE	control	RMSE	control	RMSE	control
1	+30.3	-8.6	+15.9	+29.9	+45.4	-0.4	+19.2	+7.6	+1.2	+13.5	+2.6	-43.3
2	+11.5	-50.7	-0.5	-0.7	+30.4	-36.4	+9.6	-51.4	+0.3	-132.7	+0.3	-71.4
3	+48.7	+51.0	+46.2	+70.0	+65.2	+67.3	+52.4	+42.6	+47.8	+68.2	+30.8	-1.1
4	+40.2	+36.8	+31.7	+50.9	+65.5	+61.7	+37.5	+33.6	+26.2	+34.8	+46.0	+33.2
5	+35.5	+24.4	+28.2	+46.0	+53.5	+36.6	+41.9	+19.4	+11.4	+34.3	+10.0	-32.5
6	+35.9	+57.9	+40.0	+62.1	+49.7	+73.9	+35.0	+39.0	+8.9	+35.5	+50.6	+43.1

^0^* Positive values indicate reductions relative to static control.

### A. Adaptive Closed-Loop Results

The adaptive controller was designed to address model parameter uncertainty by continuously updating the internal system representation. Fig. 2 illustrates inhibition (left) and excitation (right) results for one representative participant. On average across all participant profiles, the adaptive controller demonstrated superior tracking performance compared to the static controller, which does not estimate unknown parameters within the augmented state. Specifically, in the inhibition condition, adaptive control reduced RMSE by $ {26.9\% \pm 16.9\%}$ and total control delivery by $ {43.0\% \pm 25.5\%}$ relative to the static case. In the excitation condition, adaptive control reduced RMSE by $ {51.6\% \pm 13.2\%}$ and decreased total control delivery by $ {33.8\% \pm 43.9\%}$. These results highlight the robustness and efficiency of the adaptive approach across both inhibitory and excitatory paradigms.

The consistent improvements observed under adaptive control stem from its ability to update internal model parameters online, thereby compensating for subject-specific dynamics and time-varying physiological responses. By reducing model mismatch between the predicted and true system dynamics, the adaptive controller achieves more accurate tracking with lower corrective actions, resulting in simultaneous reductions in RMSE and total control effort. The larger relative gains observed during excitation suggest that parameter adaptation is particularly beneficial when regulating higher-arousal regimes characterized by stronger nonlinearities and greater variability.

### B. Robust Closed-Loop Results

The robust controller was designed to mitigate the impact of unmodeled disturbances and uncertainties. Fig. 3 shows representative results for inhibition (left) and excitation (right) for participant profile 1. On average across all profiles, robust control reduced tracking error relative to the static controller in both conditions. In the inhibition case, RMSE decreased by $ {16.0\% \pm 18.2\%}$ with a concomitant reduction in control effort of $ {8.9\% \pm 71.6\%}$. In the excitation case, RMSE improved by $ {23.4\% \pm 22.1\%}$; however, this improvement was accompanied by a modest increase in average control effort ($ {-12.0\% \pm 45.0\%}$), reflecting that in approximately half of the profiles, improved tracking was achieved at the expense of greater control input. Taken together, these results suggest that robust control enhances tracking accuracy but may require greater input effort, depending on the individual profile.

In contrast to adaptive control, the robust controller prioritizes stability and disturbance rejection by maintaining performance across a predefined uncertainty set rather than learning individual-specific dynamics. As a result, improved tracking accuracy can be achieved by increasing control effort, particularly during excitation, when unmodeled disturbances are more pronounced. This behavior reflects a classical robustnessefficiency trade-off, in which conservative control actions ensure reliable regulation at the potential cost of higher input demand for certain profiles.

### C. Multi-Step Transition Tracking

**Fig. 4.**
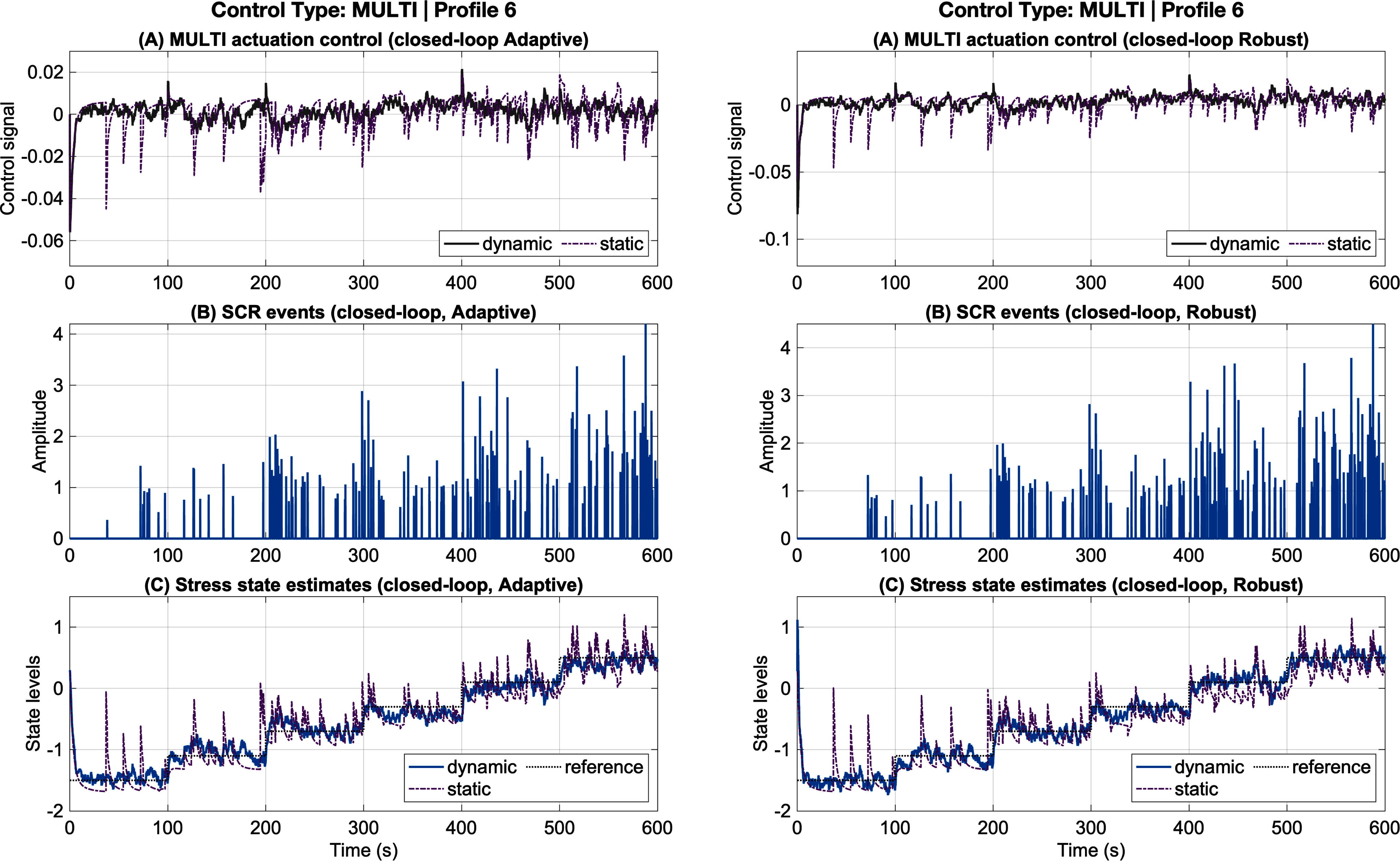
Adaptive and Robust multi-state tracking results (Profile 6). The left and right panels correspond to closed-loop adaptive and robust control systems, respectively. In each panel: (a) shows the closed-loop dynamic adaptive control input (green for inhibition, red for excitation) together with the static control (purple); (b) shows SCR events under closed-loop adaptive (left) and robust (right) control; and (e) shows the estimated stress state under closed-loop multi-state adaptive control (left) and static control (right).

To further evaluate controller performance under more complex conditions, we employed multi-step reference trajectories designed to capture gradual transitions across multiple arousal levels rather than discrete binary states. This setting provides a more challenging and realistic test of control architecture effectiveness, as it requires the system to mitigate fluctuations in the estimated arousal state in response to progressive changes in the desired latent state. Demonstrating reliable tracking in this context is critical for validating the robustness and generalizability of the proposed approaches. Panels in Fig. 4 show representative results for adaptive control (left) and robust control (right) for participant profile 1. Results for all remaining profiles are provided in the supplementary materials.

Compared to the static controller, both adaptive and robust control architectures yielded substantial improvements during multi-step tracking. On average, adaptive control achieved an RMSE reduction of $ {33.7\% \pm 12.5\%}$ and decreased total control effort by $ {18.5\% \pm 41.2\%}$. Similarly, robust control reduced RMSE by $ {32.61\% \pm 15.6\%}$ while simultaneously lowering total control effort by $ {15.1\% \pm 35.1\%}$. These results indicate that both control strategies are effective in mitigating estimated arousal states under multi-step conditions, achieving higher tracking accuracy with reduced control demands relative to the static baseline.

The comparable performance of adaptive and robust controllers under multi-step reference trajectories indicates that both approaches effectively manage gradual changes in desired arousal levels without inducing excessive corrective action. Unlike binary regulation, multi-step tracking requires sustained coordination between estimation and control to suppress transient fluctuations during state transitions. The observed reductions in both RMSE and control effort demonstrate that the proposed architectures generalize beyond discrete stressrelaxation scenarios and can support smoother, more naturalistic regulation of cognitive arousal.

## IV. Discussion

This study demonstrates that closed-loop regulation of cognitive arousal states can be quantitatively achieved using adaptive, robust control frameworks integrated with enhanced state-space models. By explicitly incorporating time-varying parameters and disturbance inputs, and using recursive Bayesian estimation to track these augmented dynamics, we extend beyond nominal, time-invariant models employed in earlier work [3], [4]. The resulting adaptive and robust controllers were both able to regulate hidden arousal states under inhibitory and excitatory conditions, validating their ability to manage inter- and intra-individual variability.

Electrodermal activity (EDA) provides a well-established and sensitive index of sympathetic nervous system activation, making it a widely used surrogate for internal arousal states [33], [34], [35], [36]. Fluctuations in EDA, particularly the occurrence and amplitude of skin conductance responses, have been consistently linked to variations in cognitive and emotional arousal [37], [38], [39]. This physiological basis supports its use as the primary marker for stress-state tracking in our framework. While additional physiological signals, such as heart rate variability, respiration, or pupil dilation, could also provide valuable insights, their integration into wearable systems often increases complexity or reduces feasibility in real-world applications [40], [41], [42]. By focusing on EDA, which can be captured unobtrusively through compact wearable sensors, we highlight a practical pathway toward scalable, real-time arousal regulation. Future work could expand this framework by incorporating multimodal physiological markers to enhance robustness.

Across both inhibitory and excitatory conditions, adaptive and robust control architectures consistently reduced tracking error relative to static operation, in which unknown parameters and disturbance inputs were not estimated. The adaptive controller achieved superior accuracy by continuously updating model parameters, thereby reducing model mismatch and enabling more precise regulation of subject-specific arousal dynamics. In contrast, the robust controller emphasized disturbance attenuation and stability guarantees across uncertainty bounds, yielding reliable tracking performance even under unmodeled perturbations, albeit at the expense of increased control effort in certain excitation profiles.

Differences between inhibitory and excitatory regulation further reflect the underlying system dynamics: excitation typically requires larger control inputs to counteract higher arousal variability and nonstationary responses, resulting in greater effort despite comparable tracking accuracy. Importantly, both control strategies demonstrated marked improvements during multi-step reference tracking, indicating an ability to manage gradual, continuous transitions in arousal rather than discrete stressrelaxation states. This capability underscores the generalizability of the proposed frameworks and their relevance for regulating naturalistic fluctuations in cognitive arousal encountered in real-world settings.

The multi-step tracking results provide additional insight into the dynamic capabilities of the proposed control architectures under more realistic regulation scenarios. Unlike binary stressrelaxation paradigms, multi-step reference trajectories require the controller to continuously coordinate state estimation and feedback control while suppressing transient overshoot and oscillations during intermediate transitions. The observed reductions in both tracking error and control effort indicate that adaptive and robust controllers effectively manage gradual shifts in desired arousal levels without resorting to aggressive corrective actions. Notably, the comparable performance of adaptive and robust designs in this setting suggests that, when reference trajectories evolve smoothly, disturbance rejection and parameter adaptation play complementary roles in maintaining regulation fidelity. These findings highlight the suitability of the proposed frameworks for regulating naturalistic, continuously varying cognitive states, where maintaining stability and smoothness across transitions is as critical as achieving endpoint accuracy.

These findings highlight the potential of closed-loop regulation for therapeutic and performance-optimization applications. For example, inhibitory control actions could trigger relaxing interventions such as music or breathing guidance [5], whereas excitatory control could provide prompts to sustain arousal when cognitive performance declines (e.g., through caffeine intake [5]). The present framework focuses on closed-loop estimation and regulation of latent cognitive arousal dynamics, treating the control input as a generic actuation channel rather than committing to a specific physical intervention. This abstraction enables a principled evaluation of adaptive and robust control strategies at the level of internal state dynamics, independent of the particular modality used to influence arousal. In practical implementations, however, any actuation capable of modulating cognitive state (e.g., sensory, behavioral, or environmental) will introduce dynamics such as subject-dependent latency, saturation, and timing constraints. Incorporating explicit models of these actuation dynamics, therefore, represents a natural next step toward experimental wearable machine-interface systems, enabling the proposed control architectures to transition from latent-state regulation to physically realizable, closed-loop interventions. Coupling machine learning and deep learning approaches with state-space modeling may further refine parameter estimation and improve characterization of actuation dynamics [43], [44], [45]. Such advances would enhance both the scalability and personalization of wearable, closed-loop systems for regulating stress and cognitive arousal. By enabling interventions to be dynamically conditioned on estimated physiological states, the proposed framework provides a principled foundation for personalized neurotechnology and precision mental health applications.

Despite these advances, the present study has several limitations that motivate future work. First, results are derived from simulation-based evaluations, and real-world deployment may introduce additional challenges such as sensor inaccuracies, environmental disturbances, and behavioral variability. Second, while electrodermal activity provides a reliable and practical marker of autonomic arousal, it represents a single physiological modality; incorporating multimodal signals such as heart rate variability, respiration, or EEG could further enhance robustness and observability of internal states [40], [41], [42]. Third, although adaptive and robust controllers were evaluated independently, future work should investigate hybrid control strategies that jointly address parameter uncertainty and unmodeled disturbances. It is important to note that the six participant profiles analyzed in this study were selected in accordance with prior work using the same dataset [3], [4], [27]. Although the original dataset included recordings from 20 individuals [25], many signals were affected by motion artifacts, range saturation, or poor skin contact, consistent with known challenges in electrodermal measurement [46]. Consequently, only six subjects provided recordings of sufficient quality for quantitative modeling and control validation. Accordingly, the reported quantitative performance metrics should be interpreted as a proof of concept, and further validation on larger and more diverse cohorts will be necessary to establish generalizability.

## V. Conclusion

This study demonstrates that closed-loop regulation of cognitive arousal states can be quantitatively achieved using adaptive, robust control architectures built on augmented state-space models. By explicitly accounting for time-varying parameters and unmodeled disturbances, both controllers substantially improved tracking performance relative to static and open-loop baselines, achieving average RMSE reductions on the order of 2550 across inhibitory, excitatory, and multi-step reference conditions.

The adaptive controller consistently delivered the largest gains in tracking accuracy, reducing RMSE by up to 52 while simultaneously lowering total control effort by approximately 3040 in both inhibition and excitation paradigms. In contrast, the robust controller reliably enhanced tracking accuracy (reducing RMSE by approximately 1633) by attenuating the impact of disturbances and model uncertainty; however, this improvement was occasionally accompanied by increased control effort in certain excitation profiles, reflecting a trade-off between robustness and input efficiency. Importantly, under multi-step transition scenarios, both adaptive and robust controllers achieved improved tracking accuracy while also reducing average control effort relative to static control, highlighting their effectiveness in regulating more realistic, gradual fluctuations in arousal.

Collectively, these results reveal complementary strengths: adaptive control excels in accuracy and efficiency under parameter uncertainty, while robust control ensures reliable tracking in the presence of unmodeled disturbances, albeit with potential increases in input demand for some individual profiles. These findings establish a foundation for wearable, closed-loop systems capable of personalized regulation of cognitive arousal for therapeutic intervention and performance optimization. Future work will focus on real-world deployment, multimodal physiological integration, and hybrid adaptiverobust control frameworks that unify learning and disturbance rejection to enable scalable, reliable, and individualized arousal regulation in everyday environments.

## Supplementary Materials

Supplementary Materials
