# Supplementary material for: Augmented State-Space Modeling and Control of Latent Arousal States Under Inhibitory and Excitatory Conditions
Source: IEEE Open J Eng Med Biol. 2026 Mar 10;7:128–38. doi: 10.1109/OJEMB.2026.3672470 (PMC13175595; doi:10.1109/OJEMB.2026.3672470)
Supplement: Supplementary Materials [file supp1-3672470.pdf]

Augmented State-Space Modeling and Control of Latent Arousal States under Inhibitory and Excitatory Conditions

Hamid Fekri Azgomi, Anan Yaghmour, and Rose T. Faghih*

I. SUPPLEMENTARY INFORMATION

FIGURES S1- S18 display the results of adaptive and robust control systems associated with Participant profiles 1-6.

Hamid Fekri Azgomi and Anan Yaghmour were with the Electrical and Computer Engineering Department at the University of Houston, Houston, TX, USA. Hamid Fekri Azgomi is now with the Department of Neurological Surgery at the University of California San Francisco, San Francisco, CA, USA. *Rose T. Faghih is with the Department of Biomedical Engineering, Tandon School of Engineering, New York University, NY, USA (correspondence e-mail: rfaghih@nyu.edu).

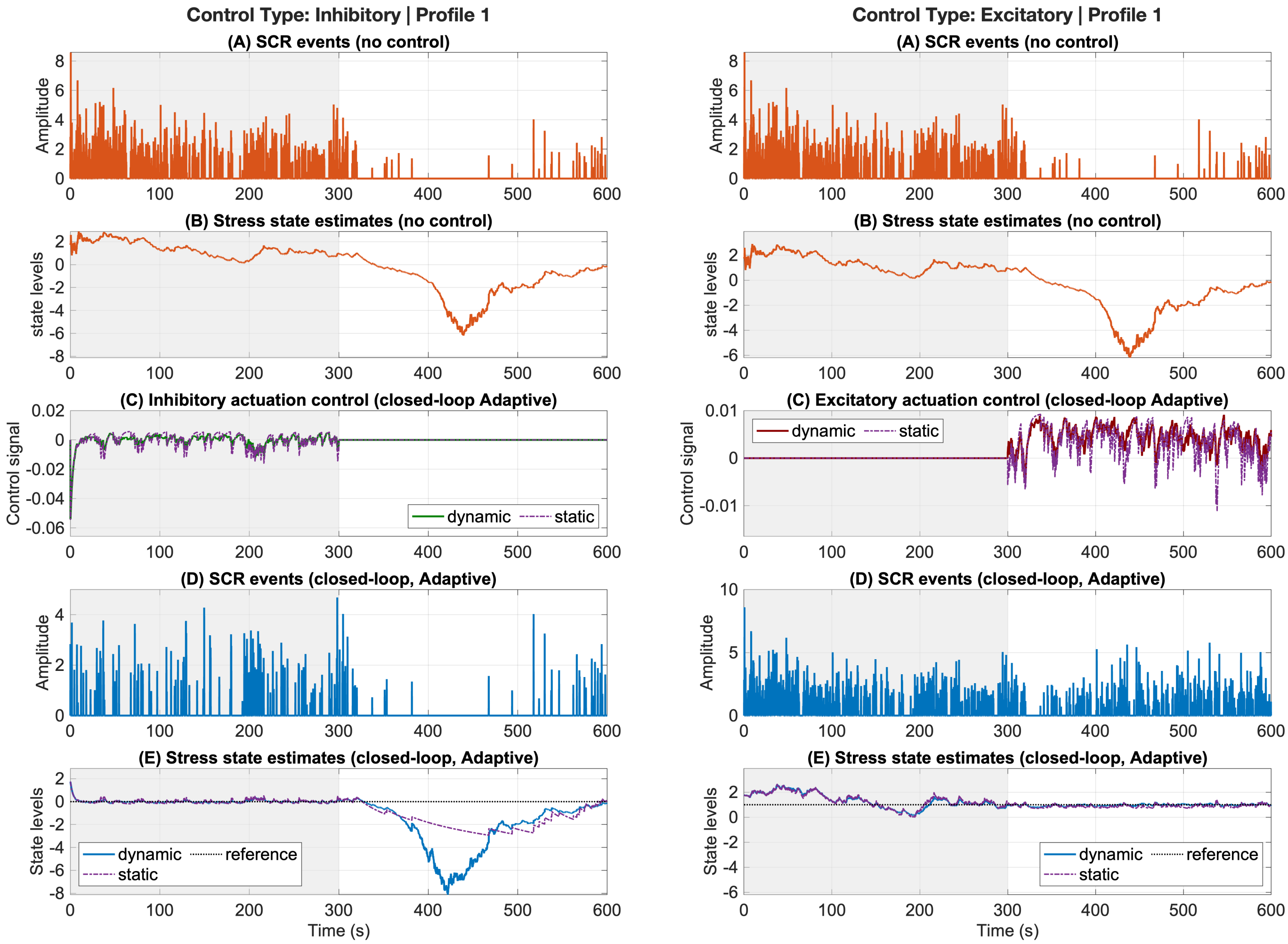

Fig. S1. **Adaptive inhibition and excitation results (Profile 1).** The left and right panels correspond to inhibition and excitation, respectively. In each panel: (A) and (B) show SCR events and estimated stress state under no control; (C) shows the closed-loop dynamic adaptive control input (green for inhibition, red for excitation) together with the static control (purple); (D) shows SCR events under closed-loop adaptive control; and (E) shows the estimated stress state under closed-loop adaptive control (blue) and static control (purple). Grey and white backgrounds denote high- and low-arousal environmental stimuli, respectively.

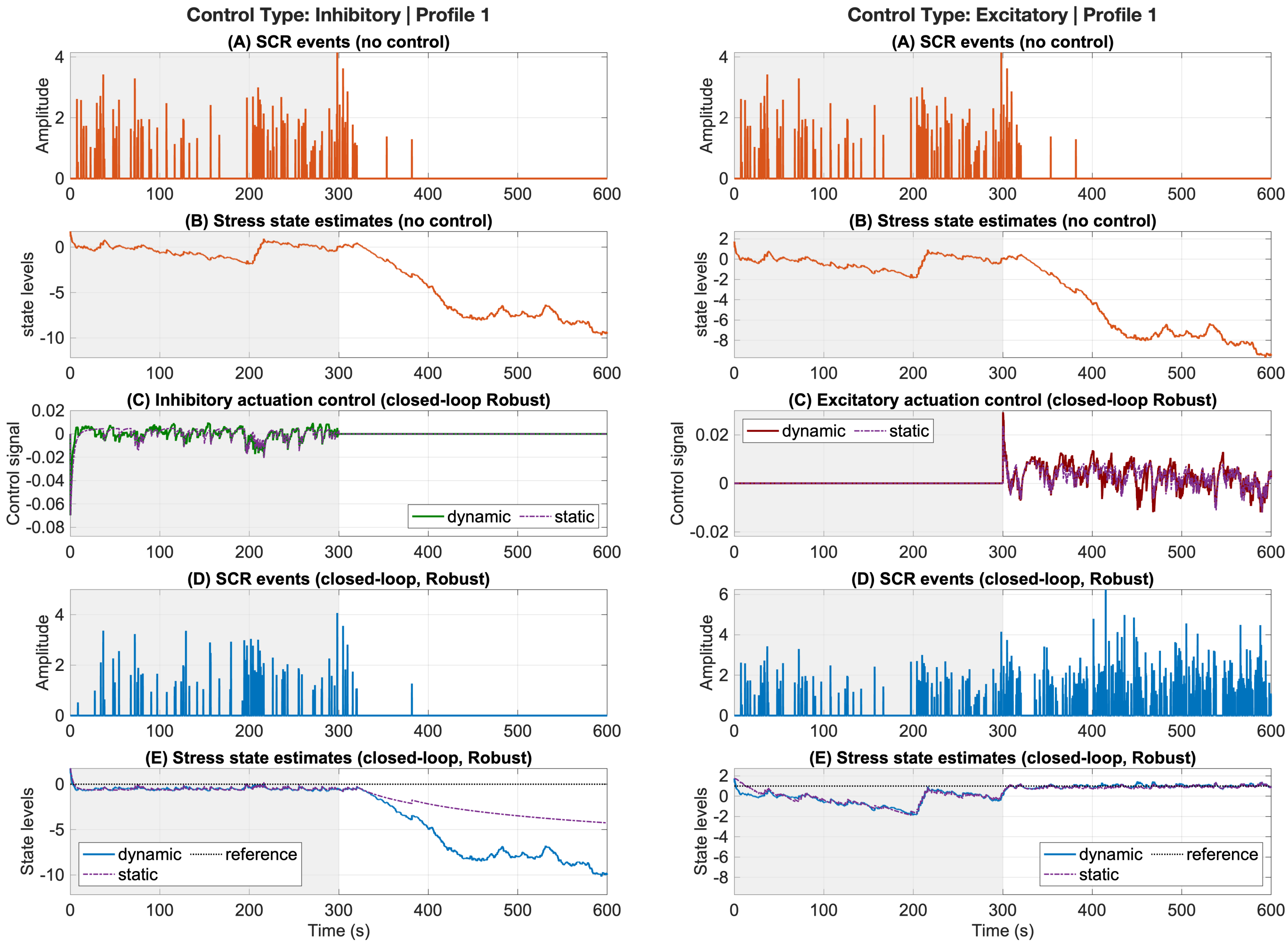

Fig. S2. **Robust inhibition and excitation results (Profile 1).** The left and right panels correspond to inhibition and excitation, respectively. In each panel: (A) and (B) show SCR events and estimated stress state under no control; (C) shows the closed-loop dynamic robust control input (green for inhibition, red for excitation) together with the static control (purple); (D) shows SCR events under closed-loop robust control; and (E) shows the estimated stress state under closed-loop robust control (blue) and static control (purple). Grey and white backgrounds denote high- and low-arousal environmental stimuli, respectively.

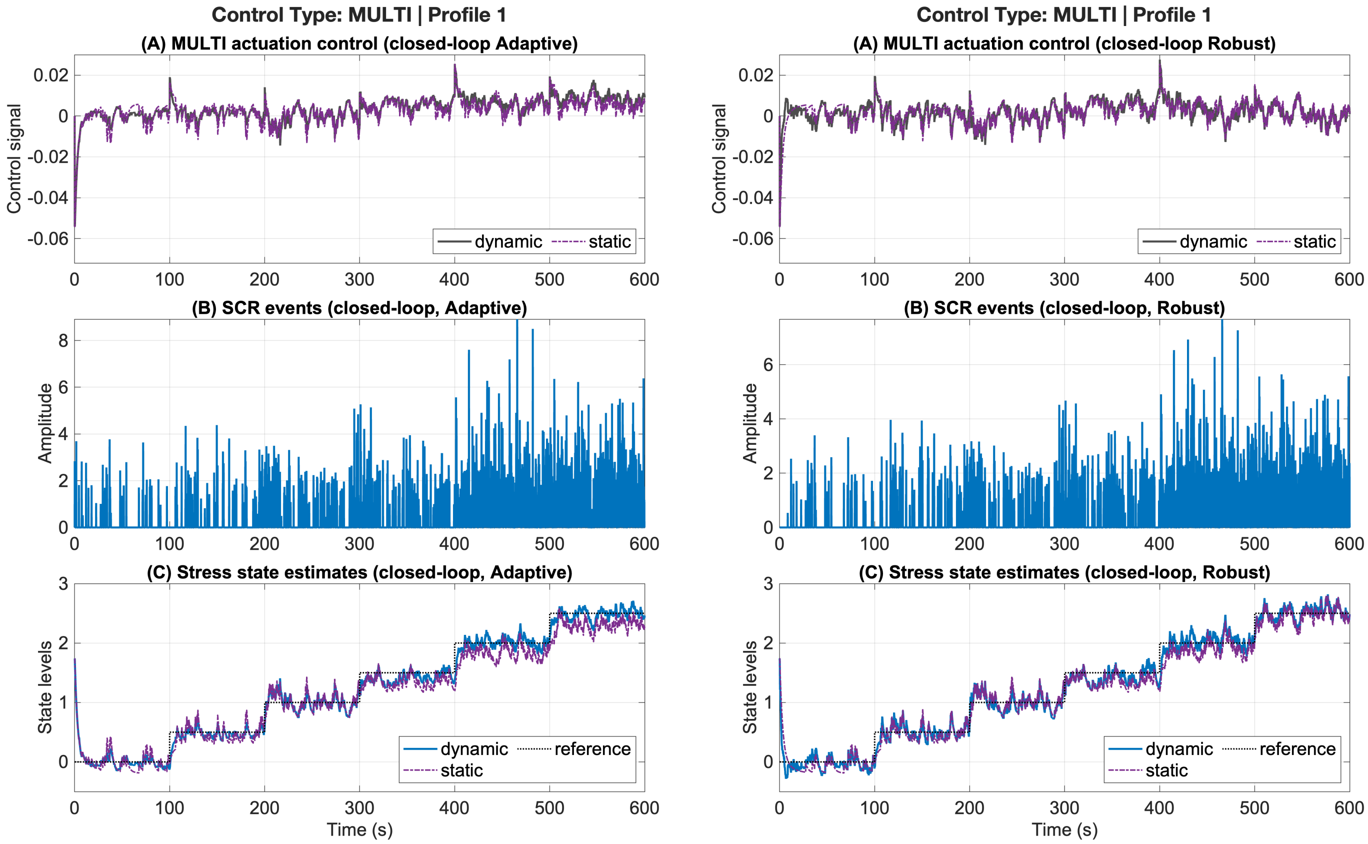

Fig. S3. **Adaptive and Robust multi-state tracking results (Profile 1).** The left and right panels correspond to closed-loop adaptive and robust control systems, respectively. In each panel: (A) shows the closed-loop dynamic adaptive control input (green for inhibition, red for excitation) together with the static control (purple); (B) shows SCR events under closed-loop adaptive (left) and robust (right) control; and (E) shows the estimated stress state under closed-loop multi-state adaptive control (left) and static control (right).

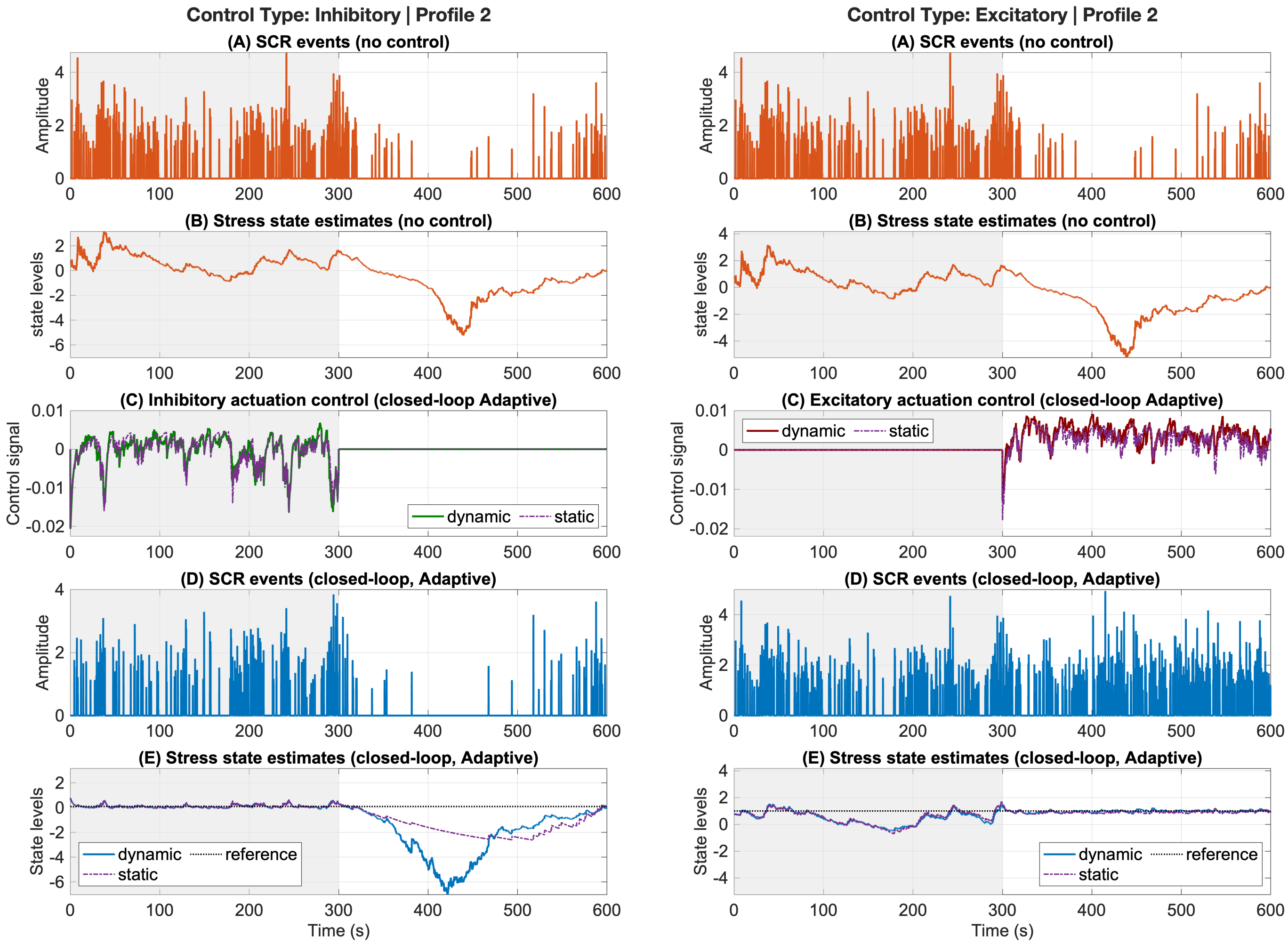

Fig. S4. **Adaptive inhibition and excitation results (Profile 2).** The left and right panels correspond to inhibition and excitation, respectively. In each panel: (A) and (B) show SCR events and estimated stress state under no control; (C) shows the closed-loop dynamic adaptive control input (green for inhibition, red for excitation) together with the static control (purple); (D) shows SCR events under closed-loop adaptive control; and (E) shows the estimated stress state under closed-loop adaptive control (blue) and static control (purple). Grey and white backgrounds denote high- and low-arousal environmental stimuli, respectively.

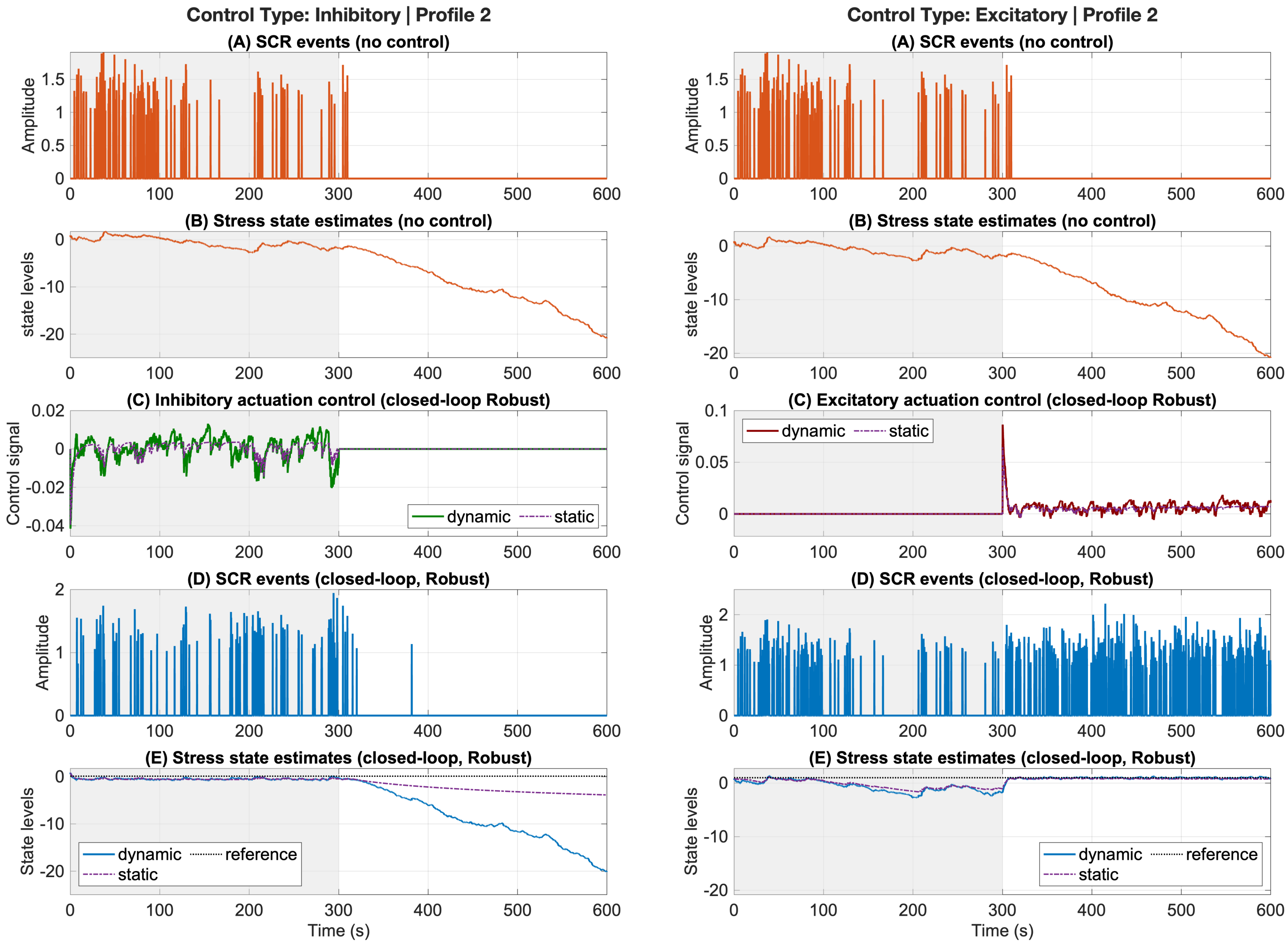

Fig. S5. **Robust inhibition and excitation results (Profile 2).** The left and right panels correspond to inhibition and excitation, respectively. In each panel: (A) and (B) show SCR events and estimated stress state under no control; (C) shows the closed-loop dynamic robust control input (green for inhibition, red for excitation) together with the static control (purple); (D) shows SCR events under closed-loop robust control; and (E) shows the estimated stress state under closed-loop robust control (blue) and static control (purple). Grey and white backgrounds denote high- and low-arousal environmental stimuli, respectively.

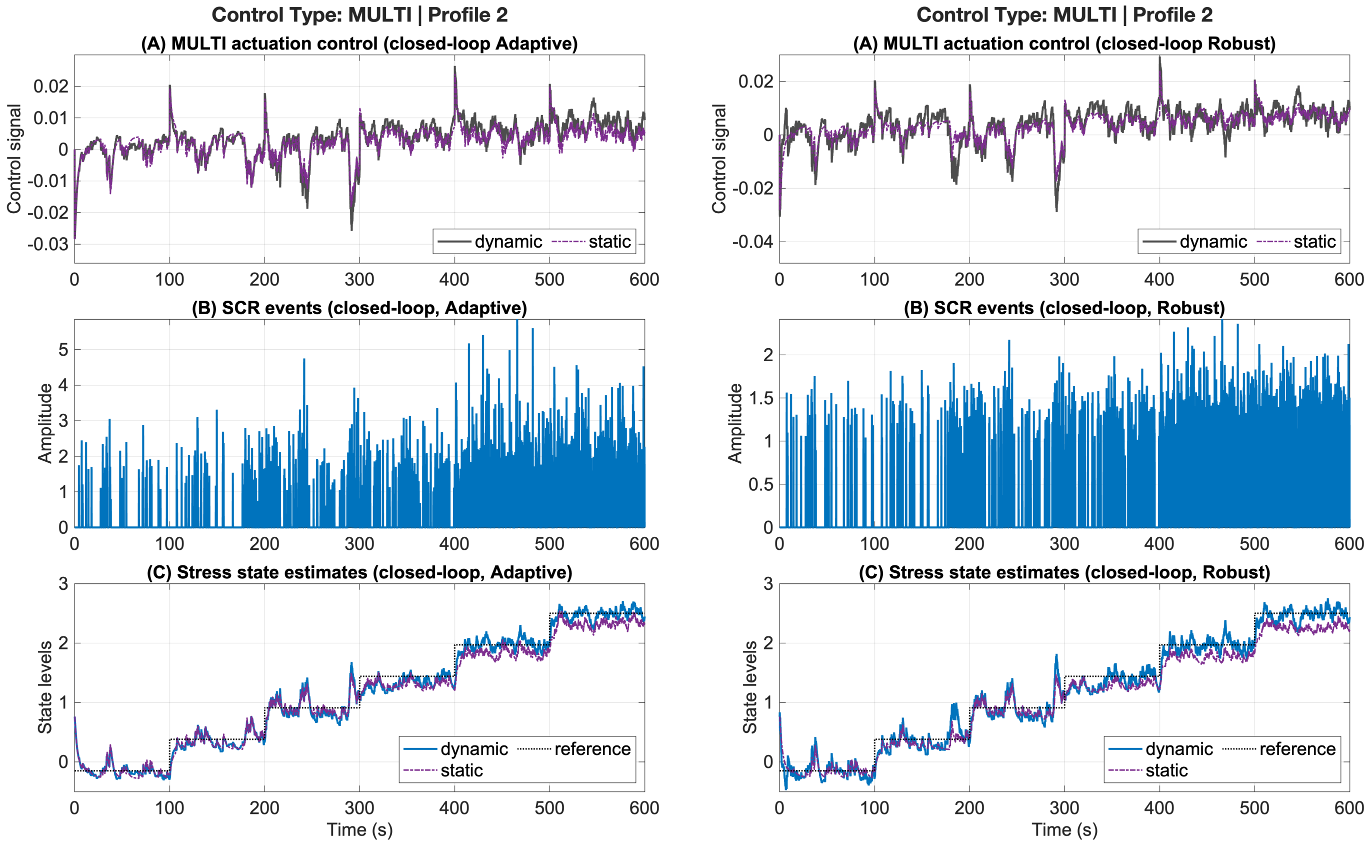

Fig. S6. **Adaptive and Robust multi-state tracking results (Profile 2).** The left and right panels correspond to closed-loop adaptive and robust control systems, respectively. In each panel: (A) shows the closed-loop dynamic adaptive control input (green for inhibition, red for excitation) together with the static control (purple); (B) shows SCR events under closed-loop adaptive (left) and robust (right) control; and (E) shows the estimated stress state under closed-loop multi-state adaptive control (left) and static control (right).

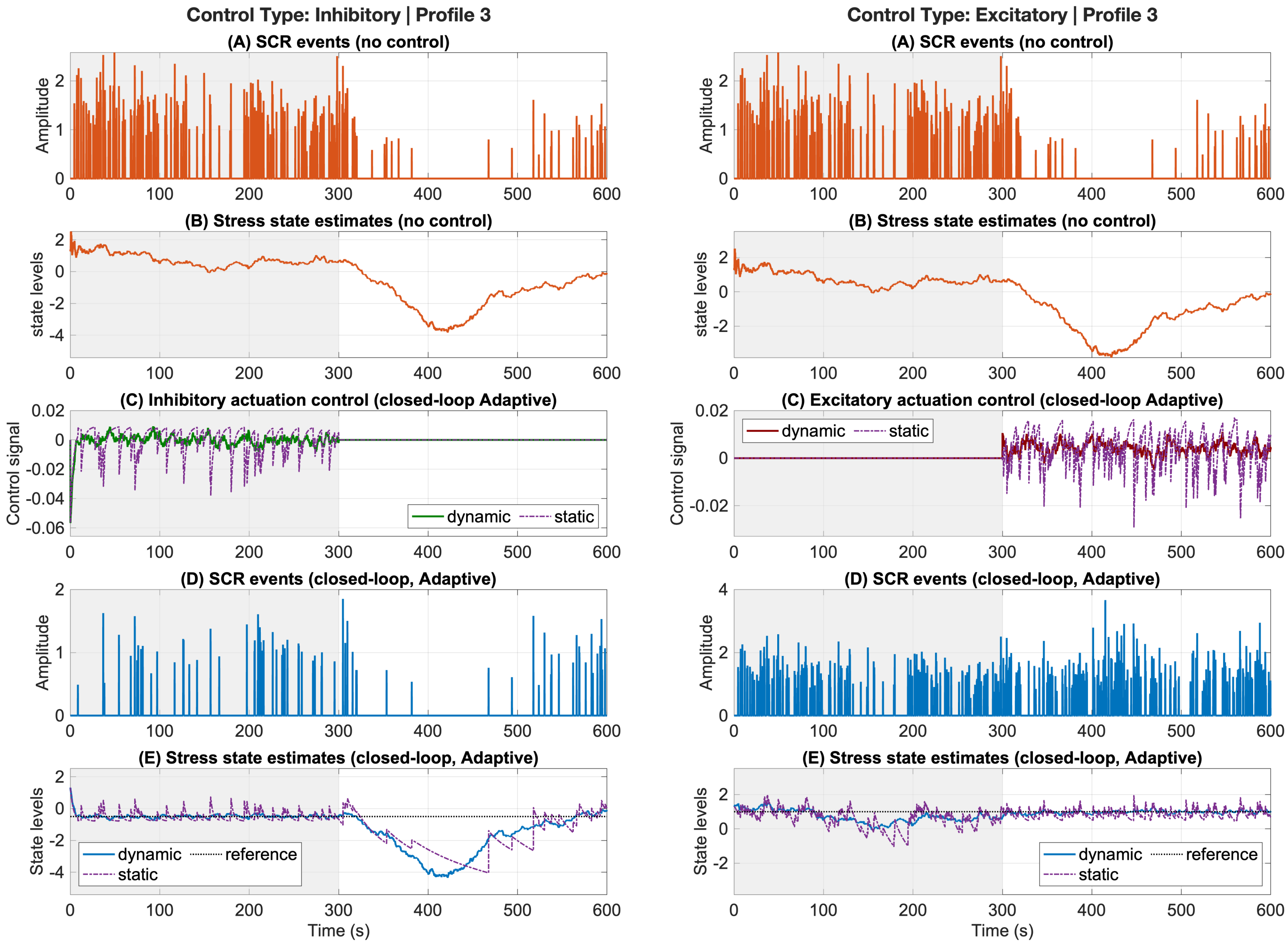

Fig. S7. **Adaptive inhibition and excitation results (Profile 3).** The left and right panels correspond to inhibition and excitation, respectively. In each panel: (A) and (B) show SCR events and estimated stress state under no control; (C) shows the closed-loop dynamic adaptive control input (green for inhibition, red for excitation) together with the static control (purple); (D) shows SCR events under closed-loop adaptive control; and (E) shows the estimated stress state under closed-loop adaptive control (blue) and static control (purple). Grey and white backgrounds denote high- and low-arousal environmental stimuli, respectively.

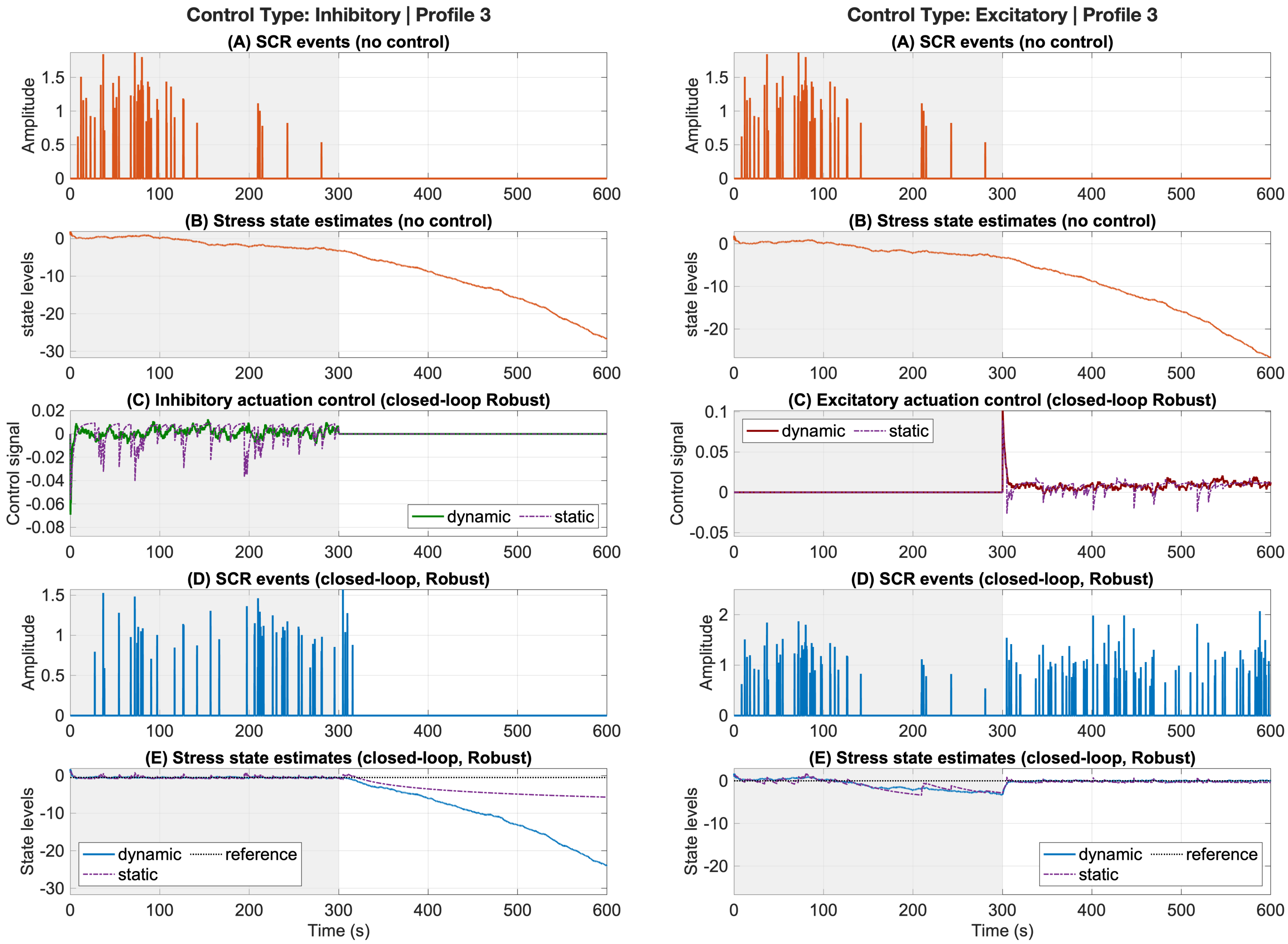

Fig. S8. **Robust inhibition and excitation results (Profile 3).** The left and right panels correspond to inhibition and excitation, respectively. In each panel: (A) and (B) show SCR events and estimated stress state under no control; (C) shows the closed-loop dynamic robust control input (green for inhibition, red for excitation) together with the static control (purple); (D) shows SCR events under closed-loop robust control; and (E) shows the estimated stress state under closed-loop robust control (blue) and static control (purple). Grey and white backgrounds denote high- and low-arousal environmental stimuli, respectively.

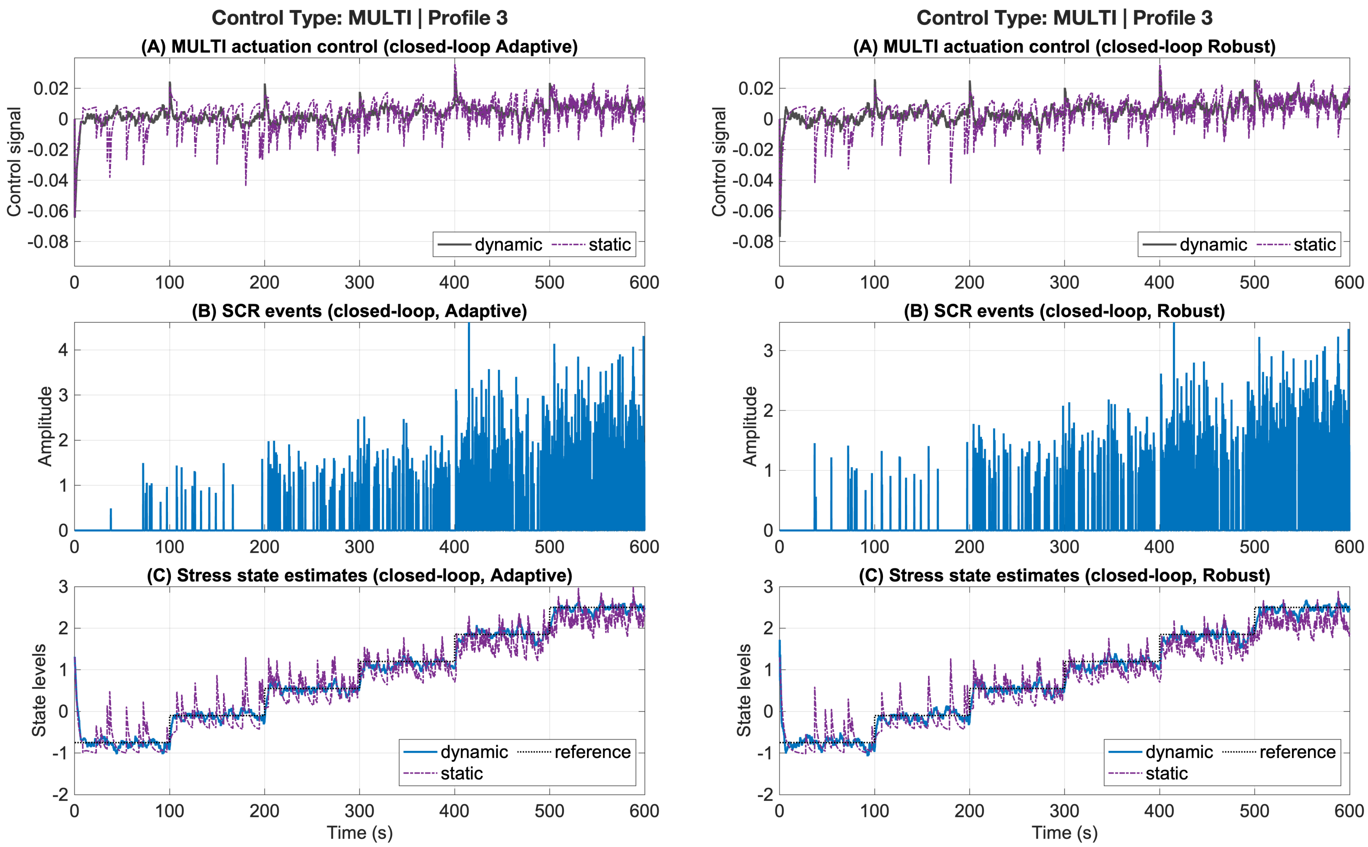

Fig. S9. **Adaptive and Robust multi-state tracking results (Profile 3).** The left and right panels correspond to closed-loop adaptive and robust control systems, respectively. In each panel: (A) shows the closed-loop dynamic adaptive control input (green for inhibition, red for excitation) together with the static control (purple); (B) shows SCR events under closed-loop adaptive (left) and robust (right) control; and (E) shows the estimated stress state under closed-loop multi-state adaptive control (left) and static control (right).

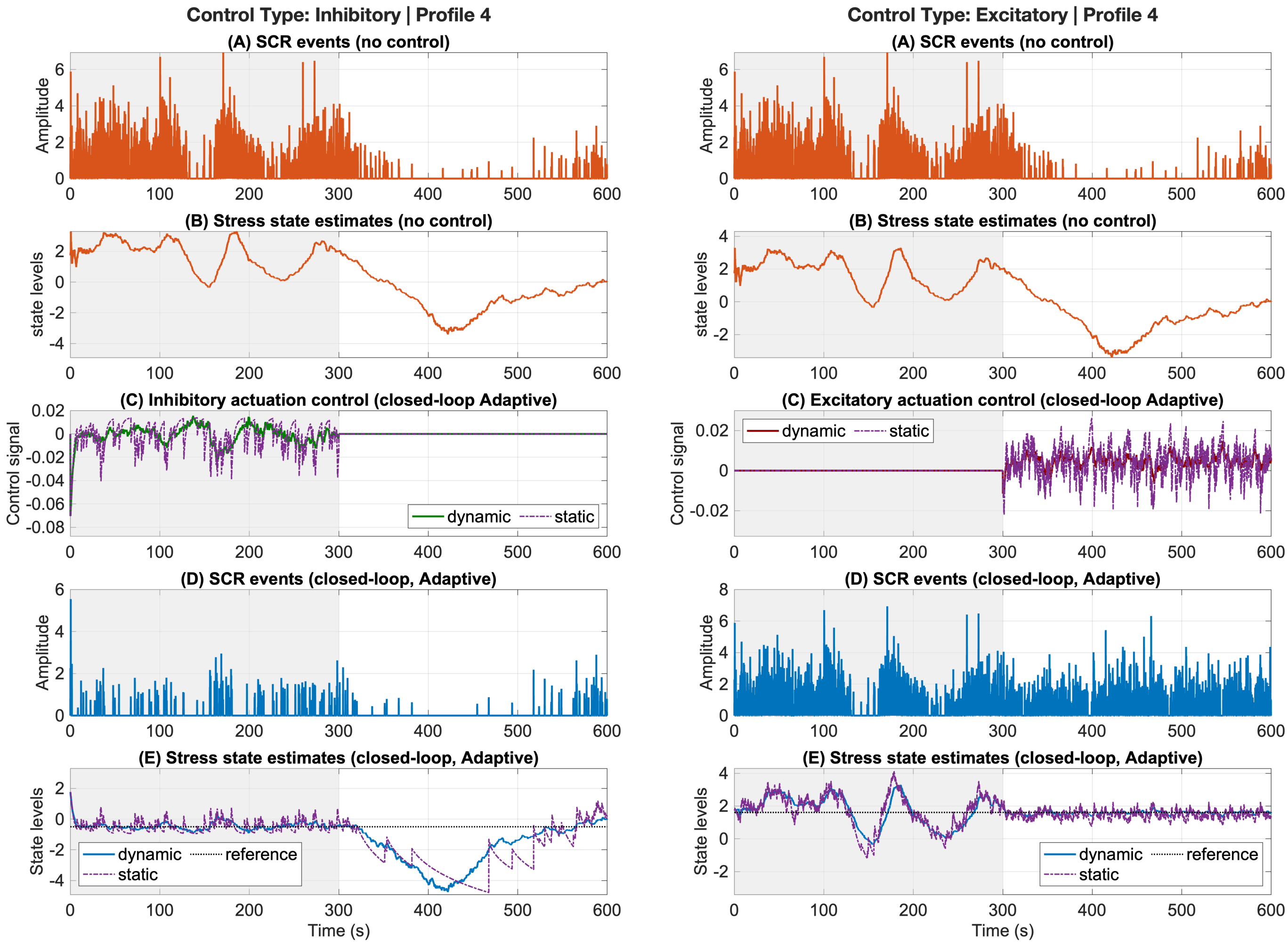

Fig. S10. **Adaptive inhibition and excitation results (Profile 4).** The left and right panels correspond to inhibition and excitation, respectively. In each panel: (A) and (B) show SCR events and estimated stress state under no control; (C) shows the closed-loop dynamic adaptive control input (green for inhibition, red for excitation) together with the static control (purple); (D) shows SCR events under closed-loop adaptive control; and (E) shows the estimated stress state under closed-loop adaptive control (blue) and static control (purple). Grey and white backgrounds denote high- and low-arousal environmental stimuli, respectively.

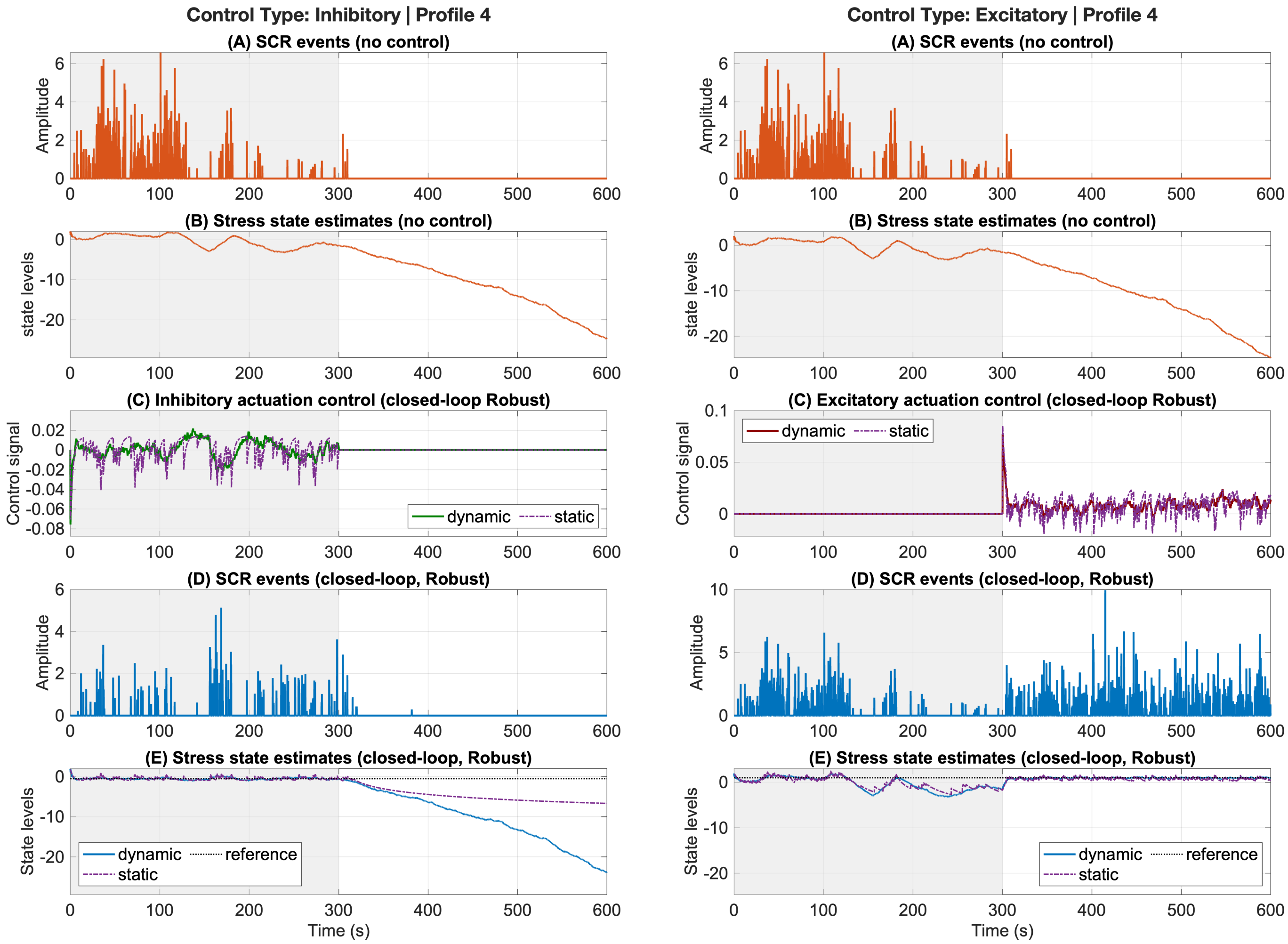

Fig. S11. **Robust inhibition and excitation results (Profile 4).** The left and right panels correspond to inhibition and excitation, respectively. In each panel: (A) and (B) show SCR events and estimated stress state under no control; (C) shows the closed-loop dynamic robust control input (green for inhibition, red for excitation) together with the static control (purple); (D) shows SCR events under closed-loop robust control; and (E) shows the estimated stress state under closed-loop robust control (blue) and static control (purple). Grey and white backgrounds denote high- and low-arousal environmental stimuli, respectively.

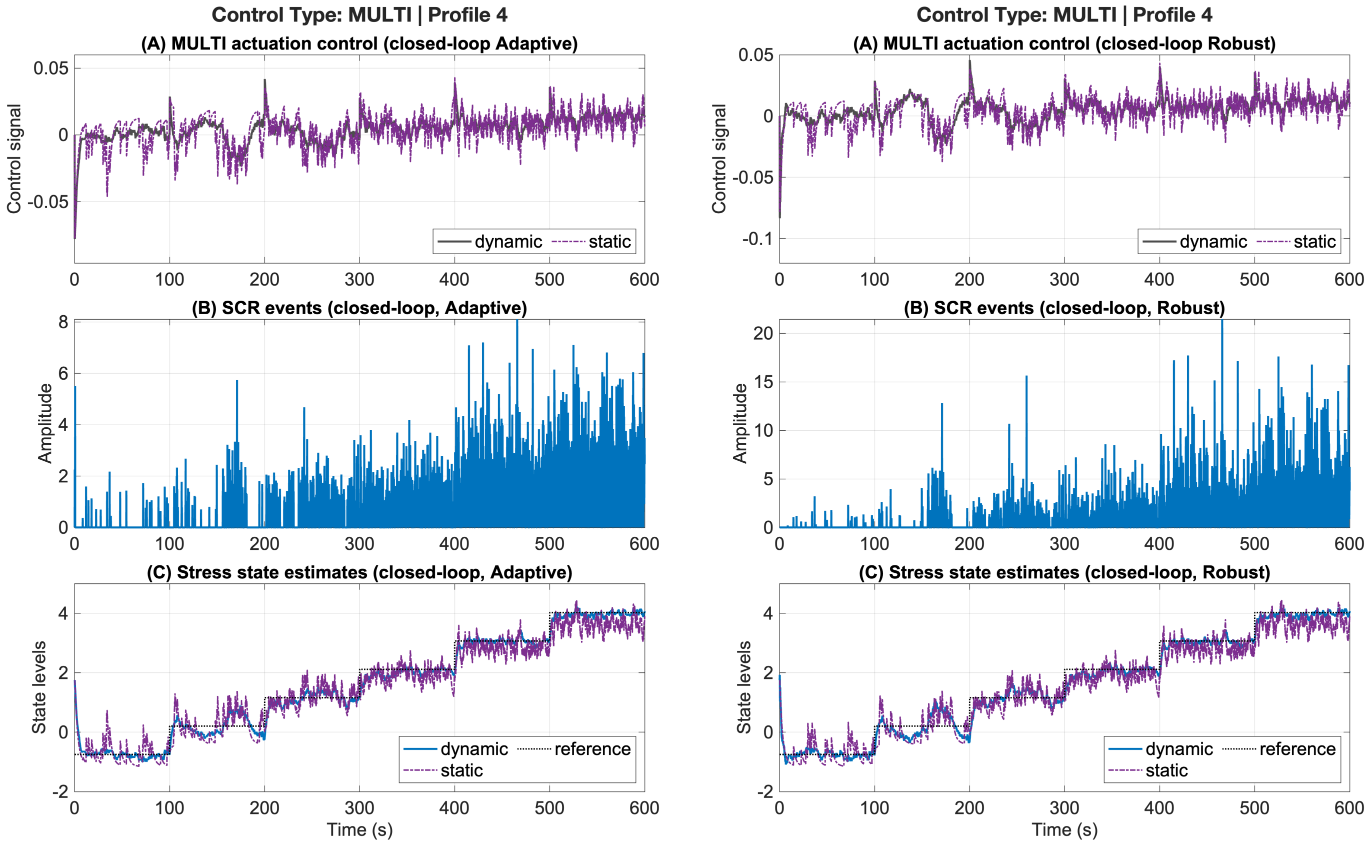

Fig. S12. **Adaptive and Robust multi-state tracking results (Profile 4).** The left and right panels correspond to closed-loop adaptive and robust control systems, respectively. In each panel: (A) shows the closed-loop dynamic adaptive control input (green for inhibition, red for excitation) together with the static control (purple); (B) shows SCR events under closed-loop adaptive (left) and robust (right) control; and (E) shows the estimated stress state under closed-loop multi-state adaptive control (left) and static control (right).

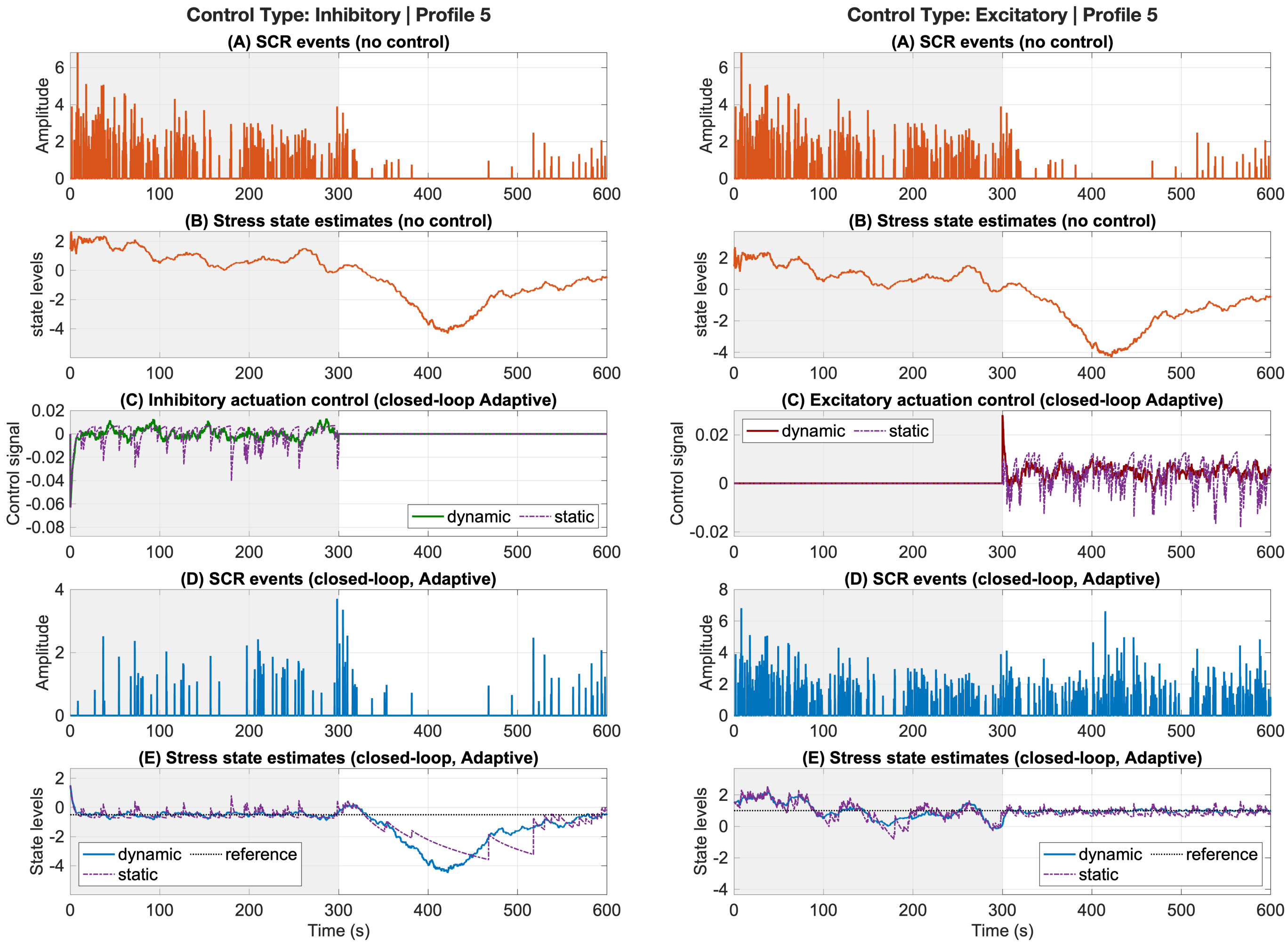

Fig. S13. **Adaptive inhibition and excitation results (Profile 5).** The left and right panels correspond to inhibition and excitation, respectively. In each panel: (A) and (B) show SCR events and estimated stress state under no control; (C) shows the closed-loop dynamic adaptive control input (green for inhibition, red for excitation) together with the static control (purple); (D) shows SCR events under closed-loop adaptive control; and (E) shows the estimated stress state under closed-loop adaptive control (blue) and static control (purple). Grey and white backgrounds denote high- and low-arousal environmental stimuli, respectively.

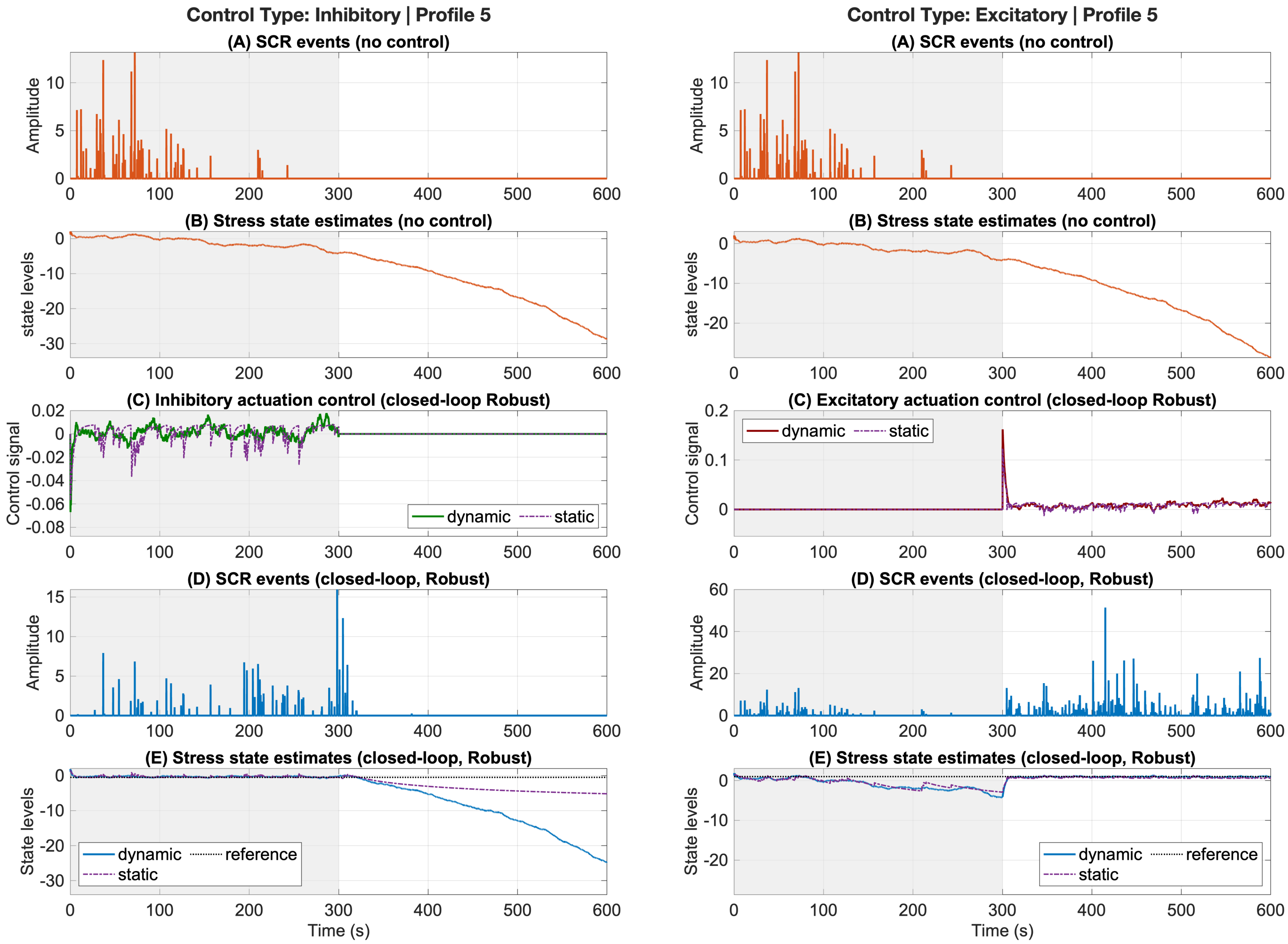

Fig. S14. **Robust inhibition and excitation results (Profile 5).** The left and right panels correspond to inhibition and excitation, respectively. In each panel: (A) and (B) show SCR events and estimated stress state under no control; (C) shows the closed-loop dynamic robust control input (green for inhibition, red for excitation) together with the static control (purple); (D) shows SCR events under closed-loop robust control; and (E) shows the estimated stress state under closed-loop robust control (blue) and static control (purple). Grey and white backgrounds denote high- and low-arousal environmental stimuli, respectively.

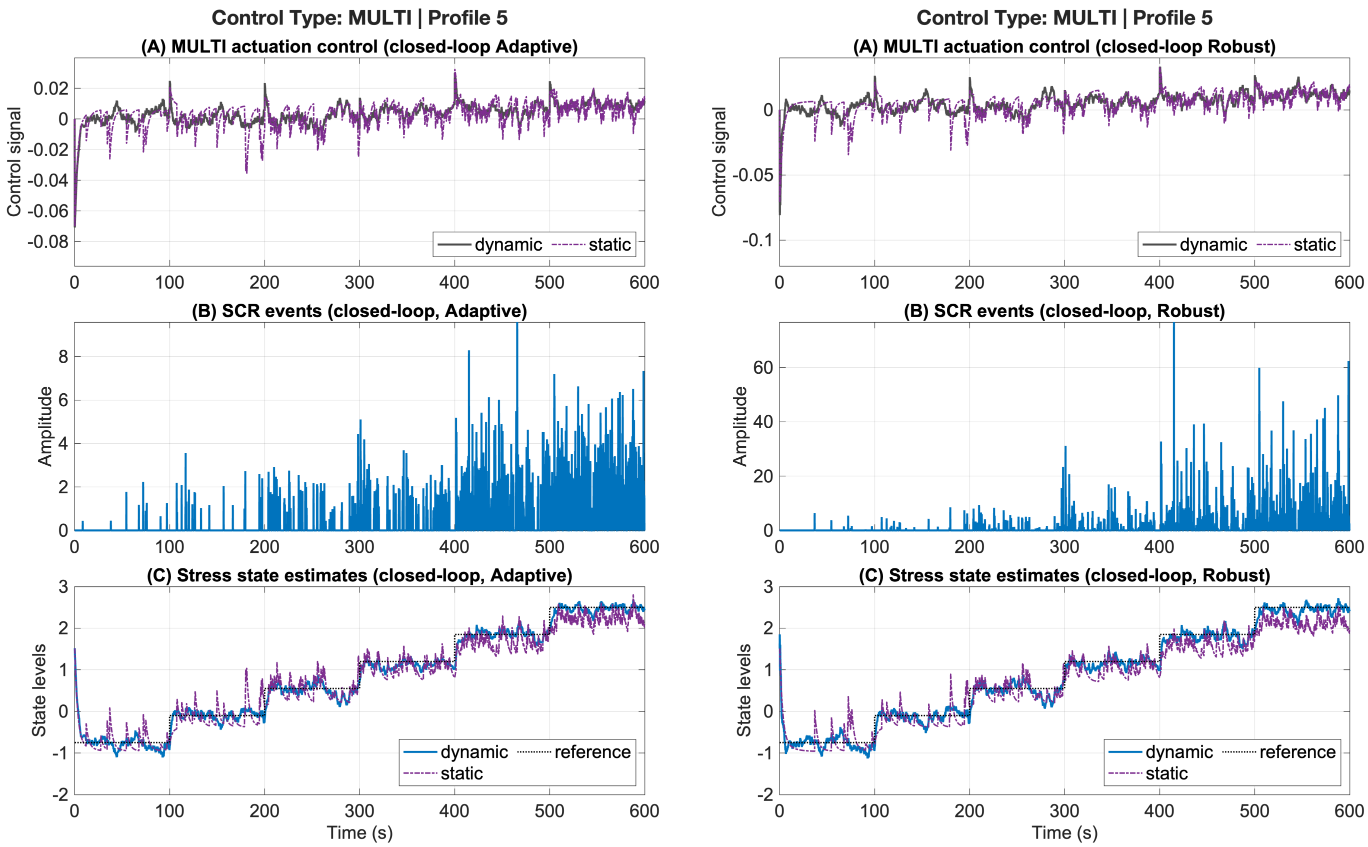

Fig. S15. **Adaptive and Robust multi-state tracking results (Profile 5).** The left and right panels correspond to closed-loop adaptive and robust control systems, respectively. In each panel: (A) shows the closed-loop dynamic adaptive control input (green for inhibition, red for excitation) together with the static control (purple); (B) shows SCR events under closed-loop adaptive (left) and robust (right) control; and (E) shows the estimated stress state under closed-loop multi-state adaptive control (left) and static control (right).

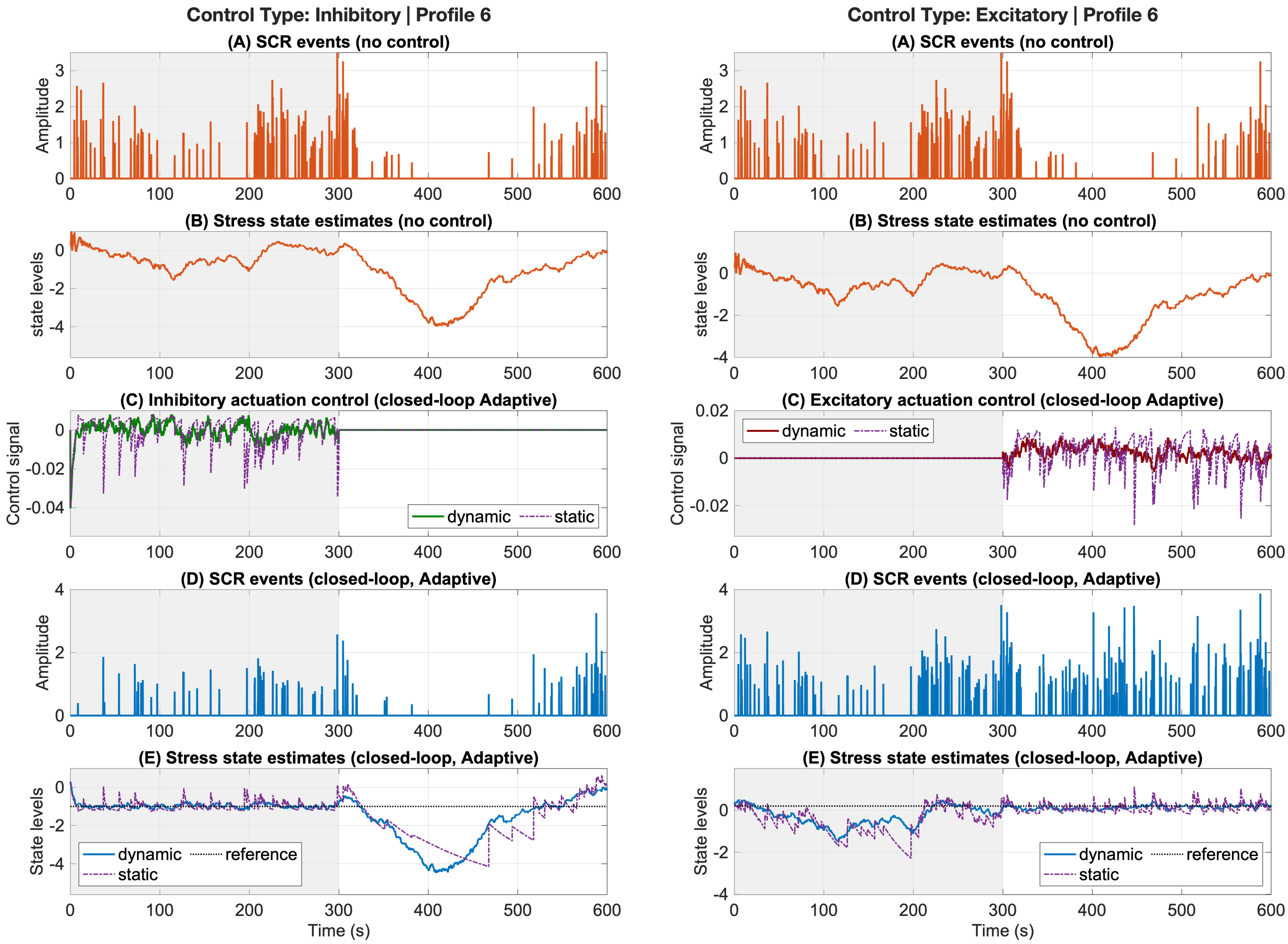

Fig. S16. **Adaptive inhibition and excitation results (Profile 6).** The left and right panels correspond to inhibition and excitation, respectively. In each panel: (A) and (B) show SCR events and estimated stress state under no control; (C) shows the closed-loop dynamic adaptive control input (green for inhibition, red for excitation) together with the static control (purple); (D) shows SCR events under closed-loop adaptive control; and (E) shows the estimated stress state under closed-loop adaptive control (blue) and static control (purple). Grey and white backgrounds denote high- and low-arousal environmental stimuli, respectively.

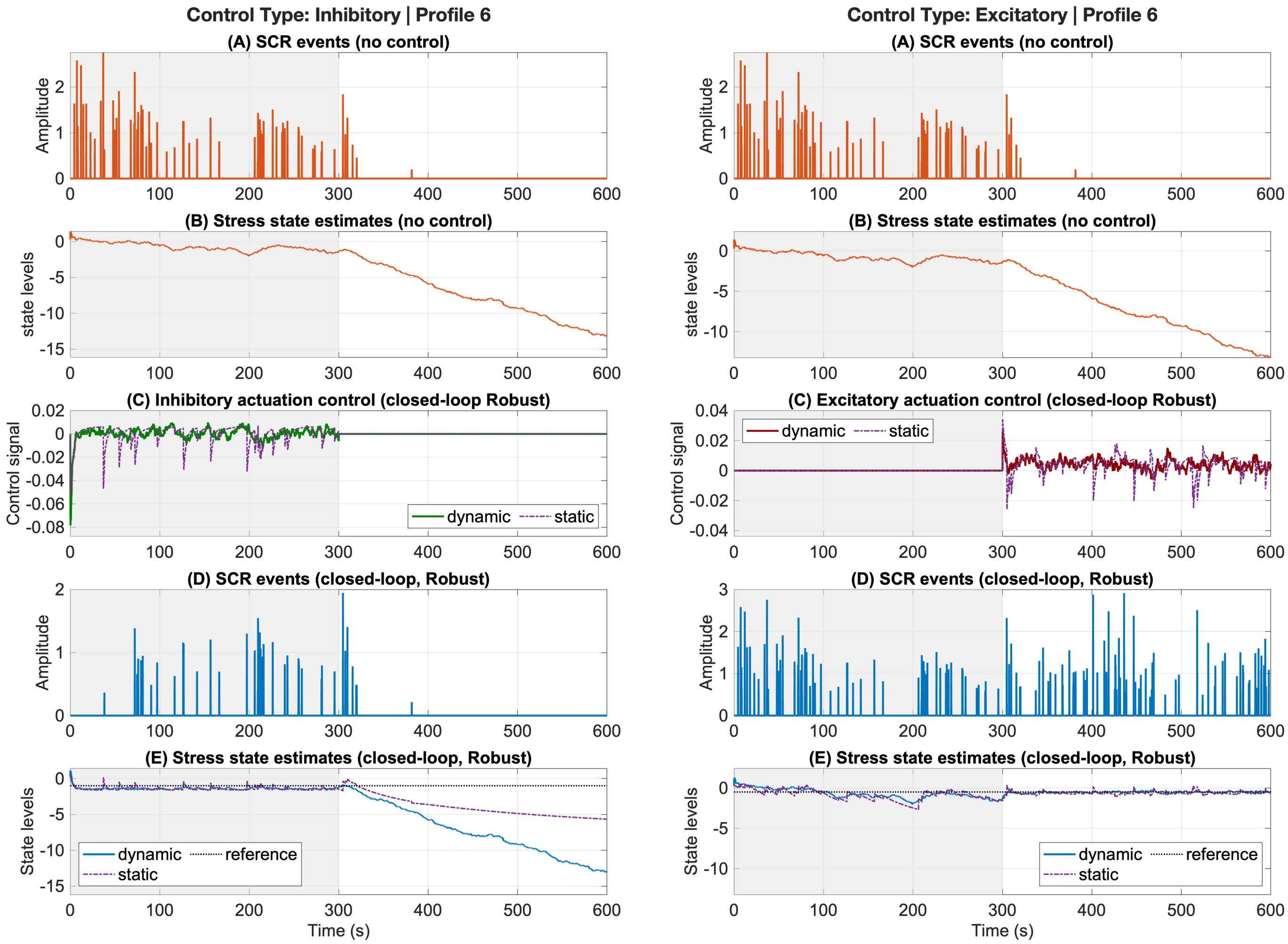

Fig. S17. **Robust inhibition and excitation results (Profile 6).** The left and right panels correspond to inhibition and excitation, respectively. In each panel: (A) and (B) show SCR events and estimated stress state under no control; (C) shows the closed-loop dynamic robust control input (green for inhibition, red for excitation) together with the static control (purple); (D) shows SCR events under closed-loop robust control; and (E) shows the estimated stress state under closed-loop robust control (blue) and static control (purple). Grey and white backgrounds denote high- and low-arousal environmental stimuli, respectively.

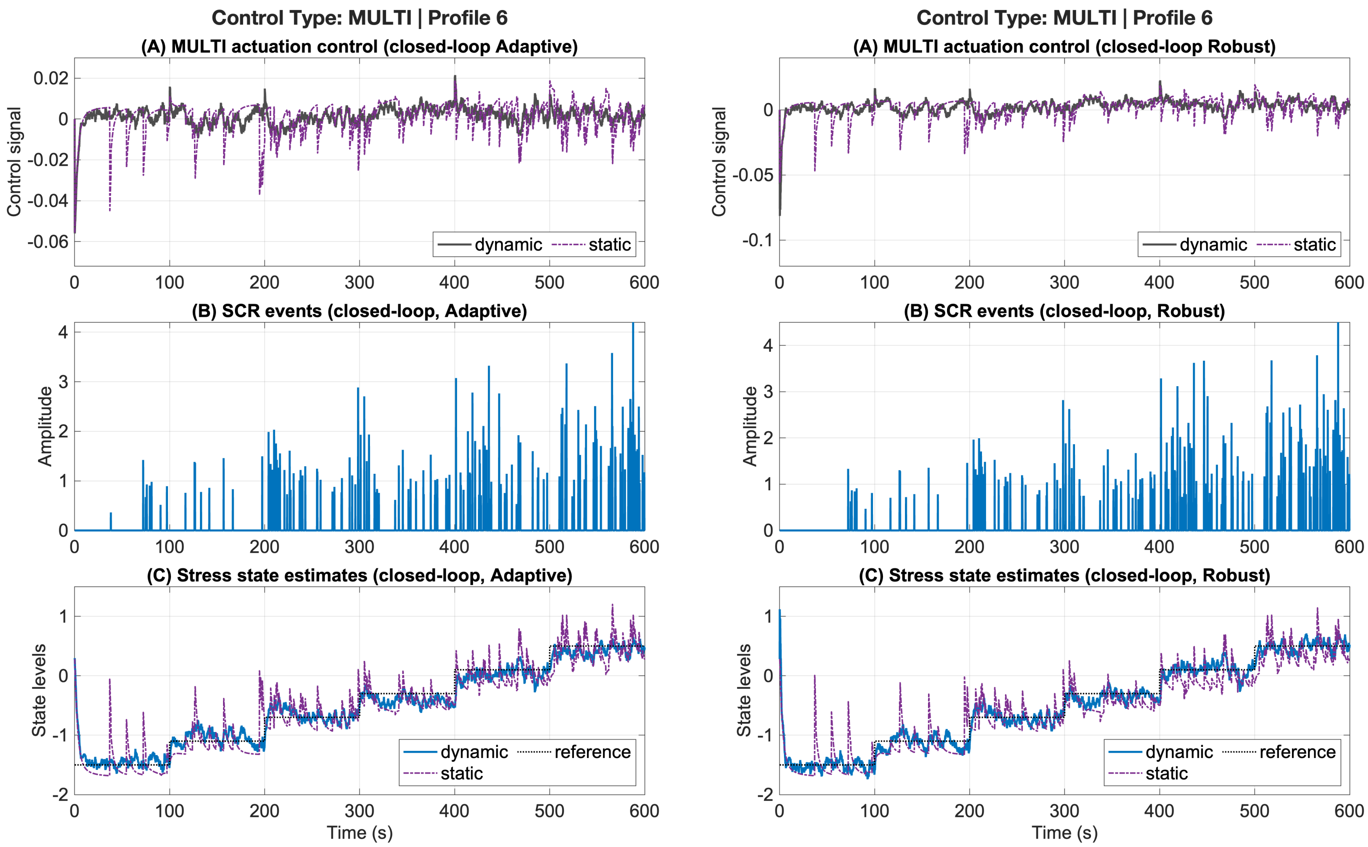

Fig. S18. **Adaptive and Robust multi-state tracking results (Profile 6).** The left and right panels correspond to closed-loop adaptive and robust control systems, respectively. In each panel: (A) shows the closed-loop dynamic adaptive control input (green for inhibition, red for excitation) together with the static control (purple); (B) shows SCR events under closed-loop adaptive (left) and robust (right) control; and (E) shows the estimated stress state under closed-loop multi-state adaptive control (left) and static control (right).
